# Systematic Review and Meta-analysis of the Impact of Chemical-Based Mollusciciding for Control of *Schistosoma mansoni* and *S*. *haematobium* Transmission

**DOI:** 10.1371/journal.pntd.0004290

**Published:** 2015-12-28

**Authors:** Charles H. King, Laura J. Sutherland, David Bertsch

**Affiliations:** 1 Center for Global Health and Diseases, Case Western Reserve University, Cleveland, Ohio, United States of America; 2 Schistosomiasis Consortium for Operational Research and Evaluation, University of Georgia, Athens, Georgia, United States of America; 3 Department of Biology, Case Western Reserve University, Cleveland, Ohio, United States of America; University of New Mexico, UNITED STATES

## Abstract

**Background:**

Programs for schistosomiasis control are advancing worldwide, with many benefits noted in terms of disease reduction. Yet risk of reinfection and recurrent disease remain, even in areas with high treatment coverage. In the search for means to better prevent new *Schistosoma* infections, attention has returned to an older strategy for transmission control, i.e., chemical mollusciciding, to suppress intermediate host snail species responsible for *S*. *mansoni* and *S*. *haematobium* transmission. The objective of this systematic review and meta-analysis was to summarize prior experience in molluscicide-based control of *Bulinus* and *Biomphalaria* spp. snails, and estimate its impact on local human *Schistosoma* infection.

**Methodology/Principal Findings:**

The review was registered at inception with PROSPERO (CRD42013006869). Studies were identified by online database searches and hand searches of private archives. Eligible studies included published or unpublished mollusciciding field trials performed before January 2014 involving host snails for *S*. *mansoni* or *S*. *haematobium*, with a primary focus on the use of niclosamide. Among 63 included papers, there was large variability in terms of molluscicide dosing, and treatment intervals varied from 3–52 weeks depending on location, water source, and type of application. Among 35 studies reporting on prevalence, random effects meta-analysis indicated that, on average, odds of infection were reduced 77% (OR 0.23, CI_95%_ 0.17, 0.31) during the course of mollusciciding, with increased impact if combined with drug therapy, and progressively greater impact over time. In 17 studies reporting local incidence, risk of new infection was reduced 64% (RR 0.36 CI_95%_ 0.25, 0.5), but additional drug treatment did not appear to influence incidence effects.

**Conclusion/Significance:**

While there are hurdles to implementing molluscicide control, its impact on local transmission is typically strong, albeit incomplete. Based on past experience, regular focal mollusciciding is likely to contribute significantly to the move toward elimination of schistosomiasis in high risk areas.

## Author Summary

Infection with *Schistosoma* blood flukes is a leading cause of chronic parasitic disease in at-risk areas of Africa, South America, Asia, and the Philippines. Over past decades, many national programs have implemented regular drug treatment to control or prevent the advanced complications of *Schistosoma* infection. However, these periodic treatments do not stop transmission of the parasite, which occurs when human sewage contaminates local water bodies and parasite eggs infect intermediate host snails. In this systematic review, we collated past experience of using chemically-mediated snail control for prevention of schistosomiasis. This approach, used in many *Schistosoma-*affected countries before the advent of the current oral drug regimens, has the potential to significantly reduce transmission if properly applied. Our meta-analysis of 63 studies (performed 1953–1981) catalogued a wide variety of water treatments and schedules employed. Among studies reporting on human infection, we found that snail control reduced local human prevalence and incidence of infection in most, but not all locations. Estimates from the aggregated studies indicate that snail control (alone) typically reduced new infections by 64% and local prevalence declined over a period of years. This decline was accelerated and more profound (84% reduction) if drug treatment was also made available.

## Introduction

Schistosomiasis, the chronic human disease caused by *Schistosoma* spp. parasite infections, is a preventable illness that, if left untreated, is associated with long-term undernutrition, anemia, organ scarring and fibrosis, resulting in disabling patient symptoms [1, 2]. Current anti-schistosomiasis chemotherapy programs focus on controlling or preventing morbidity by treating school-age children who typically have the highest levels of *Schistosoma* infection [3]. However, because pre-school infection [4–6] and recurrent infection during childhood [7, 8] are associated with significant risk for disease, optimal disease prevention can occur only when parasite infection or reinfection can be effectively blocked [9]. By themselves, Preventive Chemotherapy (PCT) campaigns [3] using mass drug administration have not been very successful in limiting transmission in high-risk areas [4, 10–13]. The WHO roadmap’s new focus on 'transmission control, wherever possible' [14] means it is appropriate to re-examine the efficacy of intermediate-host snail control for prevention of human-to-snail-to-human parasite transmission.

Reduction in infected snail numbers at the places where humans come into contact with freshwater could substantially reduce each patient's frequency of exposure to infecting parasite larvae (cercariae), and, hence, reduce the frequency of reinfection. In the 1960s early theoretical modelling [15] suggested that a greater than 90% reduction in snail numbers, in conjunction with population drug treatment, had the potential to extinguish *Schistosoma* populations from local ecosystems. Chemical molluscicides, including copper sulfate, sodium pentachlorophenate (NaPCP), N-tritylmorpholine (Frescon), and niclosamide (Bayluscide, Bayer 73) were used extensively in the 1950s, 1960s, and 1970s for schistosomiasis control in Africa, South America and Asia [16], but following the introduction of oral drug therapies, molluscicides have not seen as much use in the last 30 years [17]. As present-day programs contemplate integrated strategies for schistosomiasis control, it is important to systematically review the efficacy of molluscicide use in snail suppression and its effectiveness for infection prevention, so that planners can project likely costs and impacts when targeting parasite elimination in at-risk locations.

In the present systematic review and meta-analysis, we compiled the results of field trials of chemical mollusciciding focusing primarily on control of *S*. *mansoni* and *S*. *haematobium* species and the use of niclosamide molluscicide, now the most commonly used agent for host snail control. The decision to include just two parasite species was derived from the African focus of our sponsor, the Schistosomiasis Consortium for Operational Research and Evaluation (SCORE), and was taken in light of previous publication of a meta-analysis of mollusciciding impacts in China [18, 19]. While we encountered a number of limitations in the available study literature, we found sufficient quantitative evidence that routine mollusciciding can effectively reduce snail numbers in a manner that significantly reduces reinfection or new *Schistosoma* infection in typical at-risk human populations.

## Methods

### Ethics statement

The data used in this project were aggregated, anonymized data from previously published studies; as such, this study does not constitute human subjects research according to U.S. Department of Health and Human Services guidelines (http://www.hhs.gov/ohrp/policy/checklists).

### Study protocol and registration

The protocol for this project was developed prospectively by the authors, then registered and published in the International Prospective Register of Systemic Reviews (PROSPERO) online database, http://www.crd.york.ac.uk/prospero/index.asp, number CRD42013006869, on 16 December 2013. Our *a priori* review question was, “Does chemical mollusciciding effectively reduce snail numbers in a manner to prevent reinfection or new *Schistosoma* infection in at risk human populations?” focusing primarily on control of *S*. *mansoni* and *S*. *haematobium* species and the use of niclosamide molluscicide (2-amino ethanol salt of 2', 5'-dichloro-4'-nitro salicylanilide, sold as Bayluscide, Mollutox, and other names). The PRISMA checklist and the PROSPERO protocol for this study are provided as Supporting Information files S1 and S2 Files.

### Eligibility criteria

To quantify the effects of repetitive use of chemical mollusciciding, we aimed to include any available published or unpublished reports on its use for control of *Bulinus* or *Biomphalaria* species for prevention of *S*. *haematobium* or *S*. *mansoni* infection. No limits were placed in terms of location or language of the report. However, we did not include studies of *S*. *japonicum* or *S*. *mekongi* control, which was the topic of a recently published meta-analysis from China [18, 19]. Studies had to include periodic application of chemical compounds to transmission water contact sites or experimental locations, as well as the names of the snail species treated, and treatment doses, frequency, habitat (static *vs*. flowing water), region, and season of application. Information about local human prevalence and incidence of *Schistosoma* infection, before and after intervention, was also sought as secondary outcomes for meta-analysis. We aimed to include any studies performed after the development of niclosamide molluscicide compounds, (*i*.*e*., after January 1961) to the close of the search phase of the project, 1 January 2014. Historical perspectives, observational studies and prospective trials were eligible for inclusion if they provided the necessary quantitative data.

### Information sources

We identified published studies using PubMed, Google Scholar, Web of Science, SCIELO, African Journals Online, as well as resources such as WHO technical reports and archived files at Case Western Reserve University and the Schistosomiasis Consortium for Operational Research and Evaluation (SCORE). Where published bibliographies of the recovered studies were found to contain promising citations (including grey literature) not included in online searches, these papers were obtained, whenever possible, and screened for inclusion in the meta-analysis.

### Search strategies

We examined the available electronic database literature using combination searches of the following terms: 'molluscicide'; 'snail control/prevention'; '*Biomphalaria* (*Australorbis*)’; '*Bulinus*'; 'field [trial]' ‘schistosomiasis/prevention and control’ ‘transmission’, and/or 'niclosamide’. Secondary report finding was done by scanning PubMed 'similar articles' feature, and by using the Google- and PubMed-generated listings of papers that cited papers that we found to contain well-conducted snail control intervention trails. As relevant articles were identified, we broadened our search by accessing additional titles through the online databases’ automated ‘related articles’ links. Full titles and abstracts were recovered for the initial screening phase of study selection.

### Study selection

Review of titles and abstracts was performed by two trained reviewers, searching for data content meeting study requirements. The studies found suitable for inclusion—including historical, observational, and prospective studies—were then obtained for full-text review from online or library sources. Where a single report contained data on multiple individual community surveys, each survey was also separately abstracted for inclusion in some of the sub-group comparison analysis. We excluded studies where sufficient details of snail control measures were not reported, or when the data on the community or individual *Schistosoma* infection levels were not sufficiently detailed to confirm the reported incidence and prevalence of infection following the implementation of niclosamide or other molluscicide treatments. Cases of duplicate publication or extended analysis of previously published data were also excluded. Full listings of included and excluded studies are provided as Supporting Information files S3 and S4 Files.

### Data collection process

Included papers were abstracted and their relevant features entered into a purpose-built database created in Microsoft Excel 2013 software (Redmond, WA). These papers were archived by the authors in both paper and electronic (pdf) formats at the Center for Global Health and Diseases, Case Western Reserve University. In addition to full citation information and year of publication, information was collected on the country and region where the study was performed, along with snail genus and species, chemical mollusciciding treatment, whether the study was performed in a research laboratory or in the field, the concentration range of molluscicide delivered, and percentage kill of the observed molluscs. The effective days of molluscicide-mediated snail control were also captured, as well as the beginning and end values for population-wide incidence and prevalence. Data entries were fully verified by the second reviewer before final data analysis was carried out.

### Summary measures

Reported data on snail outcomes was quite diverse in terms of delivery, metrics, and timing of interventions and follow ups. Those results were thus summarized only qualitatively. The impact of snail control interventions on local prevalence and incidence of infection among at-risk human populations could be compared across studies, however. Results from each study and sub-study were entered into Comprehensive Meta-Analysis software, v.3 (CMA, Biostat, Englewood, NJ) for calculation of summary estimates of treatment impacts, along with their confidence intervals. Potential modifying factors assessed in preliminary analysis included parasite species, starting infection prevalence, age-group monitored, study era, region, duration of control, type of habitat, and impact of added drug treatments.

Heterogeneity among studies was expected due to differences in habitat, snail and parasite species, and human populations involved [16]. Heterogeneity levels were scored using Higgins’s and Thompson’s I^2^ statistic [20]. Summary estimates of intervention effects were computed using Der Simonian and Laird random-effects modeling [21] implemented in CMA software. The data sets used for this analysis are provided as Supporting Information files S1 and S2 Datasets. Linear meta-regression by CMA was also used to estimate the impact of duration of control on the odds/risk of *Schistosoma* infection. Potential publication bias of studies reporting impact on human incidence or prevalence of *Schistosoma* infections was assessed by visual inspection of funnel plots, and calculation of the Egger test for plot asymmetry. These funnel plots and statistics are provided as Supporting Information file S5 File.

## Results

### Study selection

**Fig 1:**
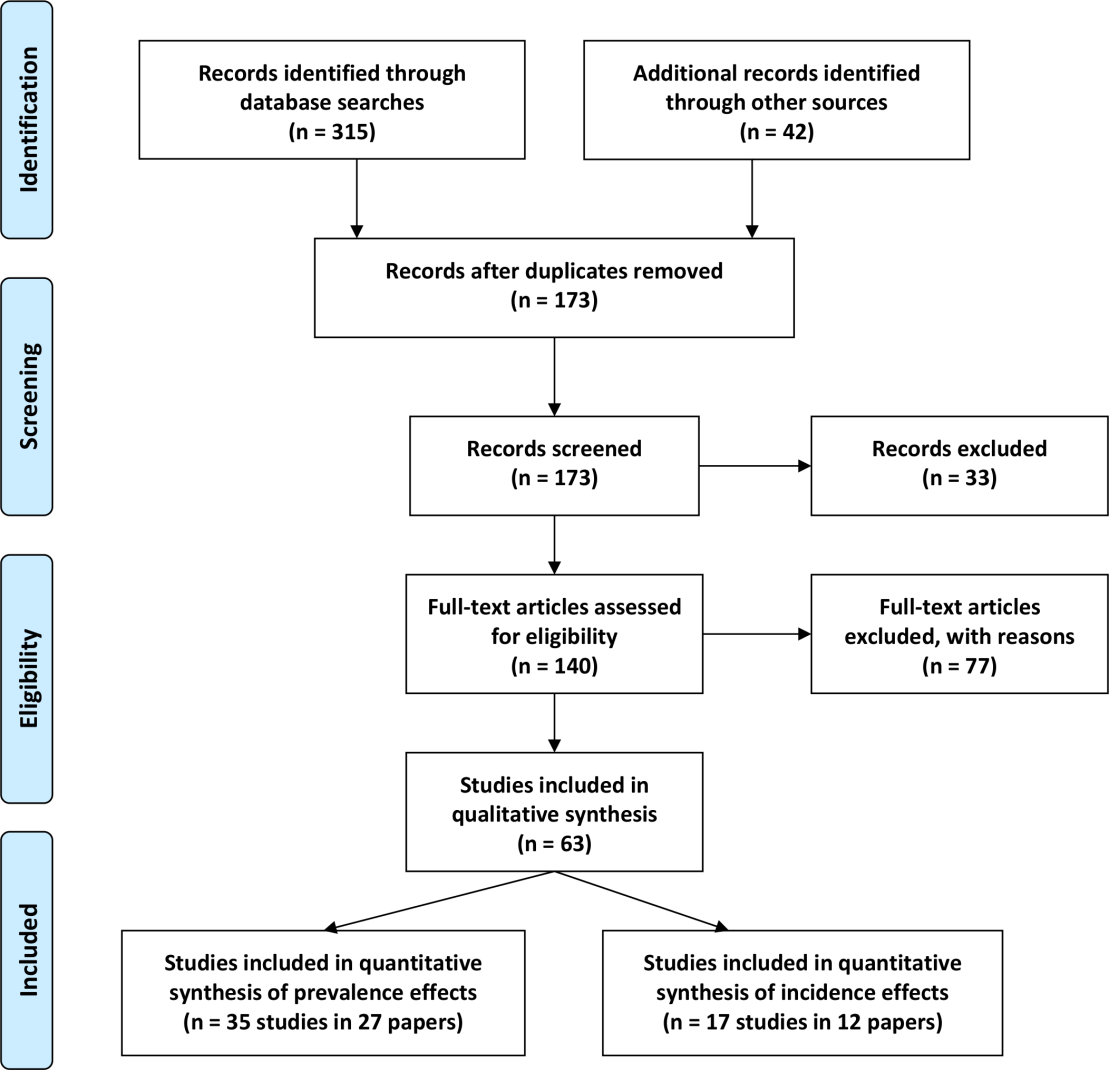
Flow chart for study selection. The flow diagram indicates the numbers of titles and studies reviewed in preparation of the current systematic review and meta-analysis of chemical mollusciciding effects on *Biomphalaria* and *Bulinus* spp. in *Schistosoma*-endemic areas.

Fig 1 contains a flow chart that details the results of the search and selection strategy for the studies included in this systemic review. Of the 357 listings recovered, 315 were obtained from online database searches, and 42 from bibliographies and archived materials. After titles and abstracts were assessed, 140 reports were selected for full review, ultimately yielding 63 studies (from 40 papers) to be included in the qualitative or quantitative analysis reported here (see Supporting Information files: S3 File ‘Listing of Included Studies’ and S4 File ‘Listing of Excluded Studies’). As detailed in the Methods section, the included studies were not limited in terms of publication date or language. The study focus was limited, however, to the control of *Biomphalaria* or *Bulinus* snail species for prevention of *S*. *mansoni* and *S*. *haematobium* transmission. Studies of *S*. *japonicum* and other human and animal schistosome species were not included.

### Study characteristics

Sixty-three reports provided information on mollusciciding’s impact on snail numbers and/or the duration of its suppressive effects. Of these, one was a graduate student thesis and the remainder were reports published in peer-reviewed journals. Twenty-seven (published) papers reported on the impact of mollusciciding campaigns on *Schistosoma* spp. prevalence among the local human population, and 12 papers provided estimates of the effects on local incidence of infection among children and/or adults. Whereas most studies reported on the use of niclosamide compounds for snail control, 6 studies used either sodium pentachlorophenate (NaPCP) [22–24] or N-tritylmorpholine (Frescon) [25] as molluscicides. For studies of niclosamide effects, publication dates ranged from 1960 to 2013 (median 1981). Of the 63 studies, 33% were from East Africa (Tanzania, Kenya, Sudan, Ethiopia), 17% were from southern Africa (Zimbabwe and South Africa), 17% were from Brazil or the Caribbean (St. Lucia), 11% were from North Africa (Egypt and Morocco), 11% were from West Africa (Gambia, Ghana, Mali), 6% were from Central Africa (Burundi, Cameroon), and 4% were from Iran.

Among 35 studies reporting impact on human *Schistosoma* spp. infection prevalence, 14 (40%) focused on prevalence among school age children, 20 (57%) reported results for the general population, and 1 (3%) reported separate results for adults and children. Eleven (31%) of these studies reported the impact of snail control as used alone, 20 programs (57%) employed snail control plus some form of community-based screening and treatment intervention (i.e., only egg-positive persons were treated), 2 studies (6%) used snail control plus a school-based treatment strategy, and 2 (6%) used snail control combined with targeted mass drug administration.

### Risk of bias

Research performed in the pre-1990s era often did not report quality-related trial details in peer-reviewed publications, and therefore, we did not pursue quality weighting in the present analysis. Many of the researchers who were involved are now deceased or retired, so access to primary data was not available. Most of the reported studies involved a single intervention site, the studies were non-randomized, and for the most part the comparison of intervention effects involved historical and not concurrent comparison data (i.e., they used a one-group, pre-test/post-test study design [26]). It is possible that the selection of study sites favored extremely high or low transmission locations. In the studies where concurrent untreated comparison sites were monitored, non-homogeneity of risk between the treated and untreated areas was often likely (e.g., Tamiem et al. [27]).

Threats to validity in assessing molluscicide-related impact on *Schistosoma* transmission included secular trends among other interventions, and maturation of environments [23, 28], heterogeneities within landscapes and populations [29], loss to follow up, and the lesser reliability of egg-count diagnostics as prevalence and intensity of *Schistosoma* infections decline [30–33]. In addition, there was potential unreliability of treatment implementation, and unknown risk of reinfection from population movement within or outside the control areas [34, 35]. The role of diffusion of information about schistosomiasis was also unknown. Observation (Hawthorne) effects were possible during implementation, particularly on plantation estate programs run by employers [36, 37]. However, in our analysis of reporting bias, we did not find evidence of underreporting of negative results or adverse outcomes (see Supporting Information file S5 File for funnel plot and regression analysis).

### Outcome results: Impact on snail numbers and duration of snail suppression

A wide variety of molluscicide delivery approaches were used in the included studies, depending primarily on the speed of water flow in the snail habitat, the extent of the water area to be treated, and the multiplicity of local human water contact sites in the area targeted for control. More rapidly flowing water bodies were often treated by drip-feed delivery systems that provided a constant dose of molluscicide to a stream or canal over a period of 1–2 days [38–41]. In irrigation schemes, where water flow could be diverted and controlled, some projects utilized impoundments of molluscicide-containing water that could be slowly transferred through the canal system to fully treat the entire irrigation system [42, 43]. Slow moving streams and static and seasonal ponds were often treated with focal spraying of shallows and vegetation at the water’s edge—the primary habitat of the intermediate host *Biomphalaria* and *Bulinus* spp. snails. Large lakes presented a special problem for treatment dosing because of rapid molluscicide dispersal by currents and wave action [44]. In one study, plastic sheeting was employed to isolate lake shore areas to retain molluscicide for a sufficient time to effect snail control [45].

Reporting on the lethal impact of molluscicide treatment and duration of its effects varied widely among studies. Early laboratory and field trials established that niclosamide concentrations of 0.1 to 3 mg/L (units equivalent to ‘parts per million’ (ppm), a term often used in the older literature) could kill over 90% of intermediate host snails [46], and that the dose effect was dependent on the duration of exposure—lower concentrations (0.04–0.53 mg/L) were effective if applied for 24h, higher concentrations (0.5–1.2 mg/L) could be lethal if applied for only 6h [47–49]. In the schistosomiasis control campaigns included in our meta-analysis, the estimated concentrations delivered to local water bodies ranged from 0.025 [37, 50] to 10 mg/L [51]. The median and most common dose target in our included studies was 1 mg/L, consonant with the experience summarized by Andrews, et al. in their detailed 1983 review [46]. Immediate impact of mollusciciding was usually assessed at breeding sites 24 h after delivery [52], and then if living snails were still present, reapplication of chemical, sometimes at higher concentrations [53], was frequently used to maximize snail suppression. Where drip feed administration was applied to running streams, significant molluscicide effects on snail numbers were detectable 900 meters [54], 1375 meters [55], 1700 meters [40], even up to 10 km [38] downstream. Snail mortality was assessed in many different ways. Some studies reported live/dead snails at various intervals after treatment, others reported on treatment impact on caged sentinel snails placed in the treated water habitats [38, 54]. Because early reapplication was used in several programs but not clearly detailed, we could not determine a summary estimate of niclosamide efficacy in terms of (dose X exposure time) [46, 47, 49, 54, 56–60] across the reported field studies. Vegetation, wave action, and debris were noted in several studies to be confounding factors affecting the molluscicidal efficacy of chemical applications [24, 44, 45, 61].

Many projects achieved 100% elimination of targeted snails for periods lasting from several weeks to several months. Some projects failed to reach 100% snail elimination following mollusciciding [35, 39, 55, 58, 61–64]. Nevertheless, these sites were able to obtain 88–99% immediate reduction in snail numbers. When snail re-emergence occurred, repopulation times ranged from 2 weeks [62] up to 18 months [65], depending on location and habitat. Serial monitoring for significant snail repopulation at human water contact sites was an important part of implementation, and was most often used to decide the intervals needed for repeated mollusciciding.

**Fig 2:**
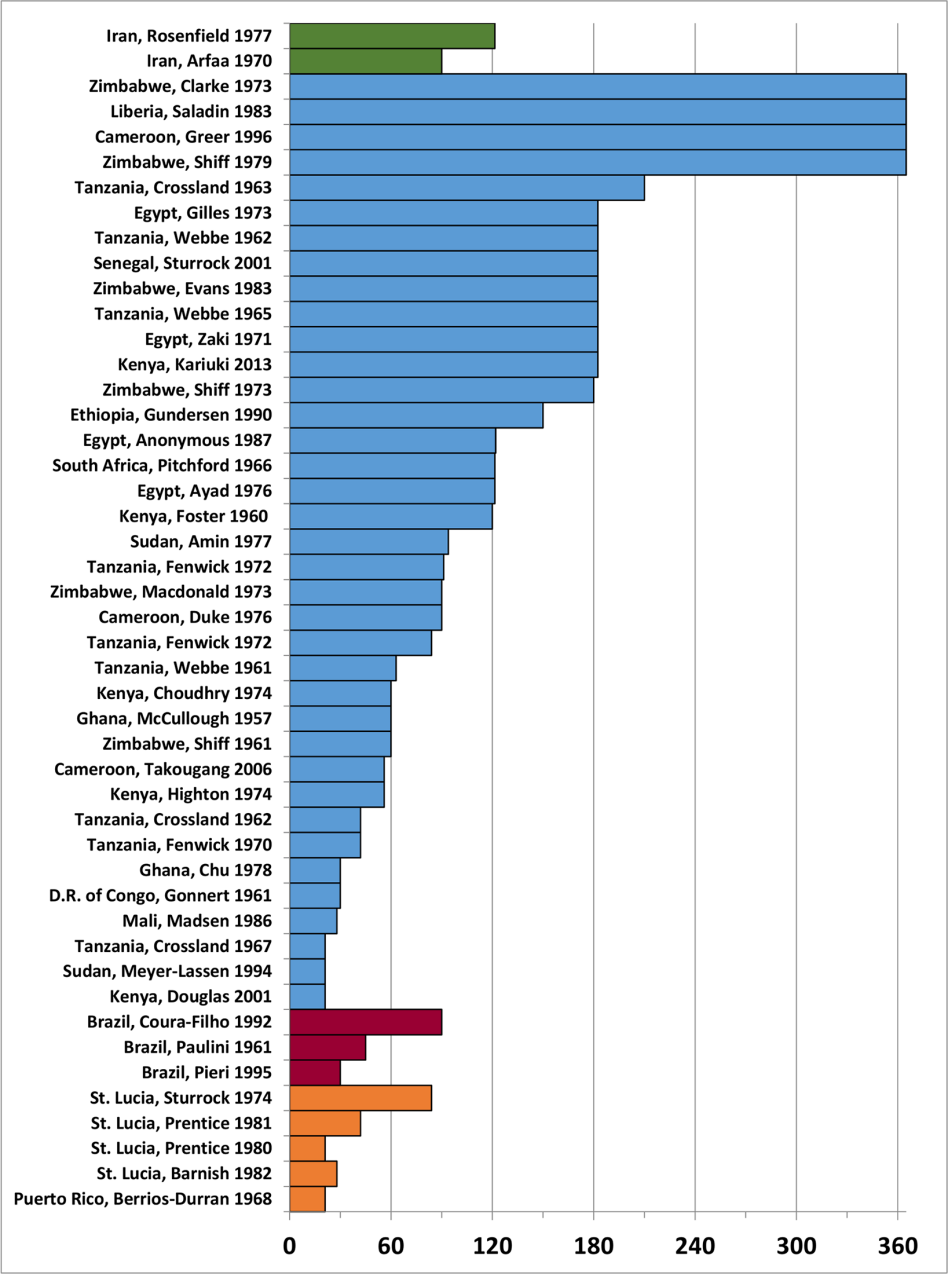
Working molluscicide treatment intervals reported by snail control programs, by region. Horizontal bars, grouped according to world region, indicate the operative between-treatment intervals (in days) used for mollusciciding in 47 different schistosomiasis control programs in Asia (green), Africa (blue), South America (red), and Caribbean locations (orange).

Fig 2 indicates the between-treatment mollusciciding intervals reported by 47 studies of *S*. *mansoni* and *S*. *haematobium* control in different areas of Asia (Iran), Africa, South America (Brazil) and the Caribbean (St. Lucia and Puerto Rico). There was a large range of working between-treatment intervals reported (21 days to 365 days), depending in part on the seasonality of transmission, the type of water treated (flowing, static, or canal), and the desired lethality of the treatment applied (suppression vs. elimination of snails). Decisions regarding treatment intervals were most often based on snail repopulation detected on regular waterside surveys at treated water contact sites. The median interval used in the reported studies was 90 days, with an inter-quartile range (IQR) of 42–90 days.

### Program impact on prevalence of human infection

**Fig 3:**
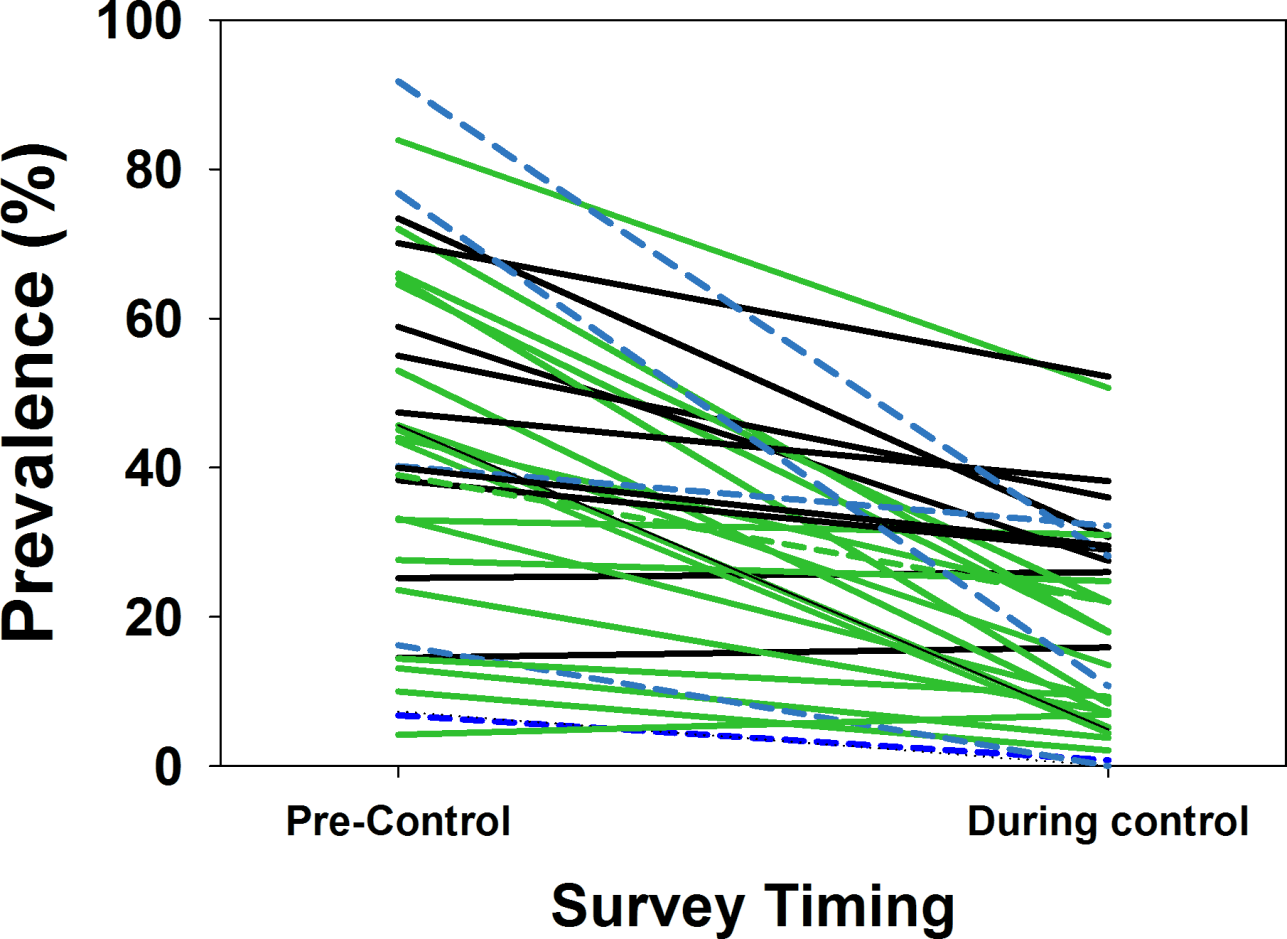
Change in local human prevalence of *Schistosoma* infection during mollusciciding control projects. Individual lines represent the shift from pre-control prevalence (left side) to prevalence after implementation of chemical mollusciciding (right side) by schistosomiasis control programs. Black lines indicate programs that used niclosamide mollusciciding alone, green lines indicate programs that combined mollusciciding with anti-schistosomal drug treatments as part of control, and the dashed blue lines indicate studies where non-niclosamide molluscicides were utilized.

**Fig 4:**
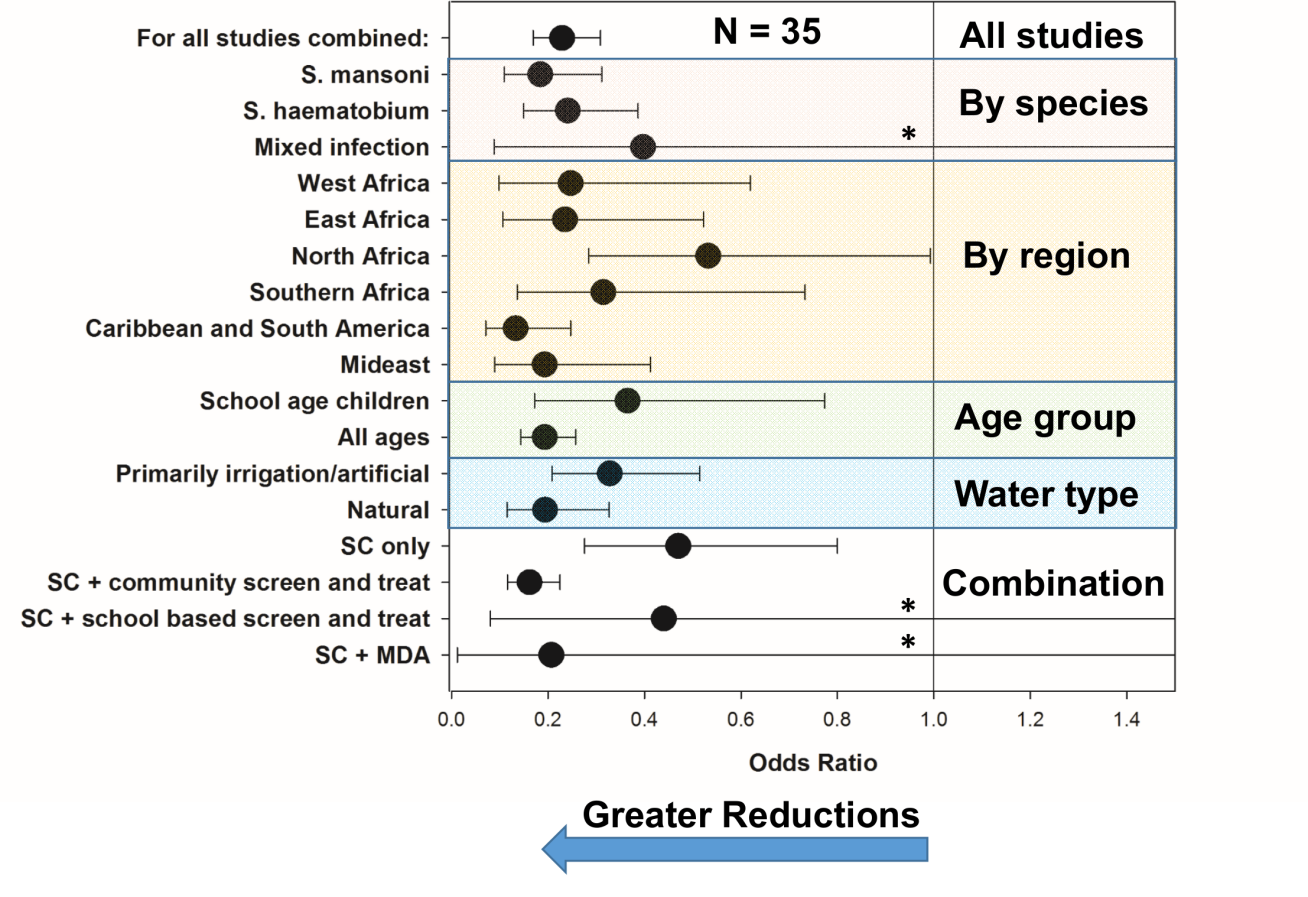
Odds ratios for *Schistosoma* infection, as measured by human prevalence, after implementation of mollusciciding programs. Black circles indicate odds ratios estimated by random effects meta-analysis for *Schistosoma* infection prevalence after mollusciciding, as compared to pretreatment levels. Whisker bars indicate the 95% confidence interval for the OR estimates. Asterisks (*) indicate that the graphing of the upper confidence limit was truncated for that category (numeric details are provided in Supporting Information file S1 Table). Results are shown for all studies reporting human prevalence data before and after control (top line, N = 35), and for study subgroup categories classified according to parasite species, geographic region, which age groups were included, the types of water bodies treated, and whether the program included snail control (SC) only, or mollusciciding combined with some form of drug treatment intervention.

Thirty-five studies reported in 28 publications [8, 22–24, 27, 28, 34, 35, 39, 41, 42, 66–81] reported on the interval impact of programs that included snail control campaigns on the prevalence of detectable *Schistosoma* infection among local human populations. Heterogeneity in prevalence outcomes was quite high (I^2^ = 99.9) among studies. Fig 3 indicates the pre- and post-intervention levels of *Schistosoma* prevalence for individual studies; the median pre-control prevalence for all studies was 45%, (Range: 5% to 92%, IQR 26% to 58%) while the median post-control prevalence was significantly lower, 17.5% (Range: 0% to 53%, IQR 6% to 30%, (*P* < 0.001 by Wilcoxon signed rank test)). Supporting Information file S1 Fig shows the forest plot for the prevalence studies. Fig 4 graphs summary estimates of the odds ratio for infection after molluscicide treatment intervention as compared to pre-treatment levels (numeric details for this graph are provided in Supporting Information file S1 Table). Overall, surveyed populations had a significantly reduced odds of infection (OR 0.23, CI_95%_ 0.169, 0.309) following snail control intervention. The impact was less strong where snail control was used alone (OR 0.47, CI_95%_ 0.276, 0.800), and greatest among studies where snail control was combined with community-based screening and treatment programs (OR 0.162, CI_95%_ 0.116, 0.225). There was not a significant difference in terms of impact between *S*. *mansoni-* and *S*. *haematobium*-endemic locations. Treatment of natural water sites had greater overall impact than treatment of irrigation systems, which may account for the observation that North Africa (primarily irrigation locations) had less improvement than sites elsewhere in Africa, Asia, South America, and the Caribbean. Studies focused only on school age children reported lesser gains in terms of post-intervention prevalence when compared to general population studies, likely reflective of school age groups’ much greater risk for infection/reinfection. Not shown, starting prevalence of infection did not have a clear effect on the size of prevalence reductions obtained during a mollusciciding program (for details, see Supporting Information file S3 Fig, which shows a forest plot of the range of outcomes data (ORs) arranged according to starting prevalence of *Schistosoma* infection).

Of note, three (9%) of the 35 studies [66, 77, 81], two in Egypt and one in Zimbabwe, did not demonstrate reductions in local *Schistosoma* prevalence. In addition, another three studies reported less than a five percentage point drop in local human *Schistosoma* prevalence during their mollusciciding trial period [24, 39, 73]. These three less successful studies were performed in Egypt and in Liberia and involved both *S*. *mansoni* and *S*. *haematobium* areas. A summary of the implementation, population, and environmental features of these six projects having relatively limited mollusciciding impact is included in Supporting Information file S2 Table. Their individual reports provided several possible explanations for their limited program impact. These included i) having only a short duration of follow-up (i.e., 1 year after mollusciciding implementation) [24]; ii) a lesser impact of supplemental drug treatments on *S*. *mansoni* as compared to *S*. *haematobium* [39, 77]; iii) inability of the implemented molluscicide program to reduce snail numbers at transmission sites [66, 81]; and iv) incorrect timing of mollusciciding application relative to maximal seasonal transmission [73].

**Fig 5:**
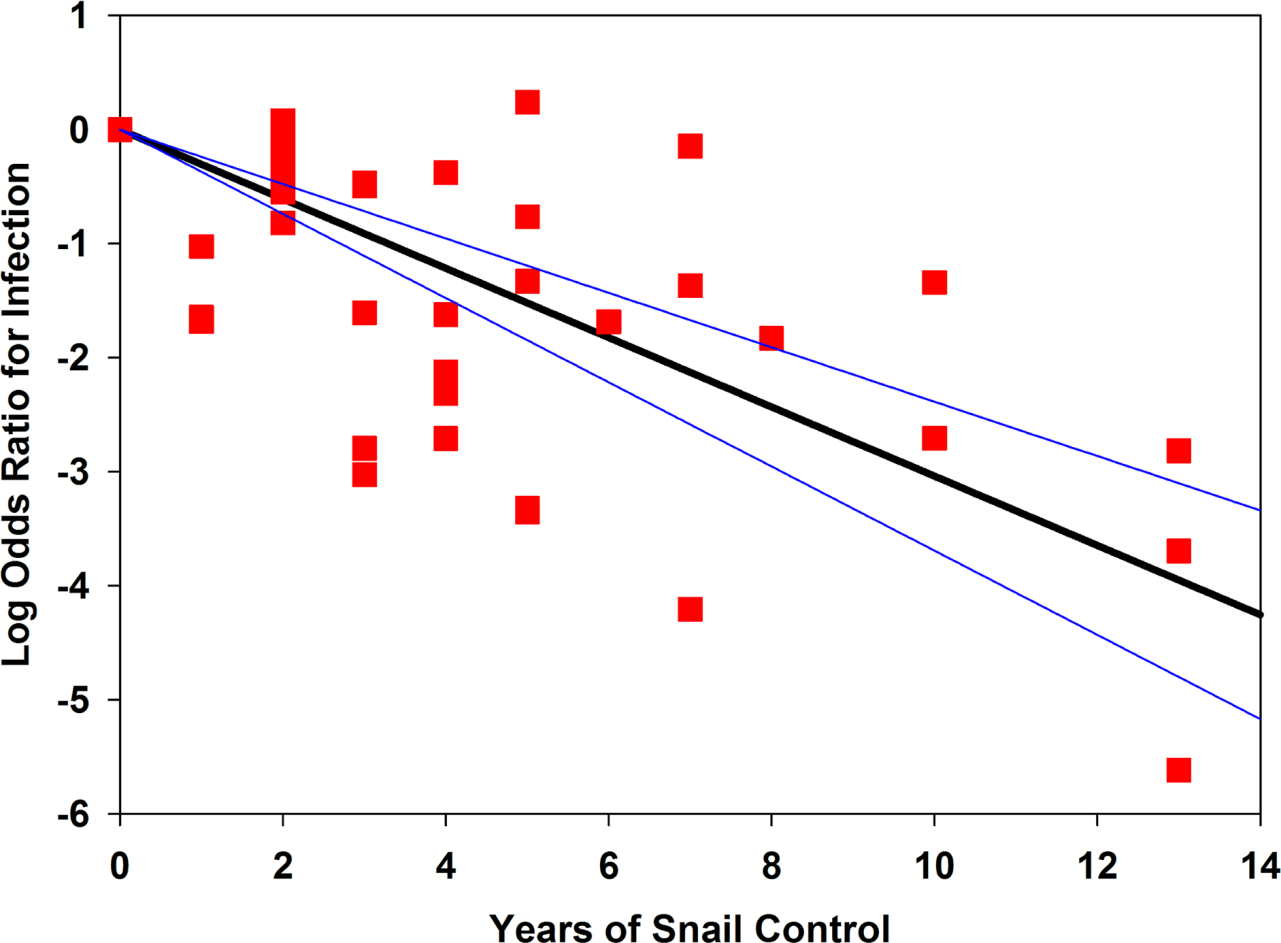
Meta-regression of *Schistosoma* infection prevalence log-odds ratio according to the duration of mollusciciding control. Red squares indicate the point estimates for individual studies, graphed as the log odds ratio of infection after mollusciciding (y axis) against the reported duration of snail control (x axis). The dark line indicates the best-fit regression line for the combined studies, anchored at zero effect at time 0. The thin lines indicate the 95% confidence band for the regression.

In consideration of the long-term effects of multi-year programs, a meta-regression of mollusciciding effects on prevalence odds ratios vs. time is presented in Fig 5. It suggests progressively greater reductions in infection prevalence as mollusciciding programs extend beyond the first few years of snail control.

### Program impact on incidence of human infection

**Fig 6:**
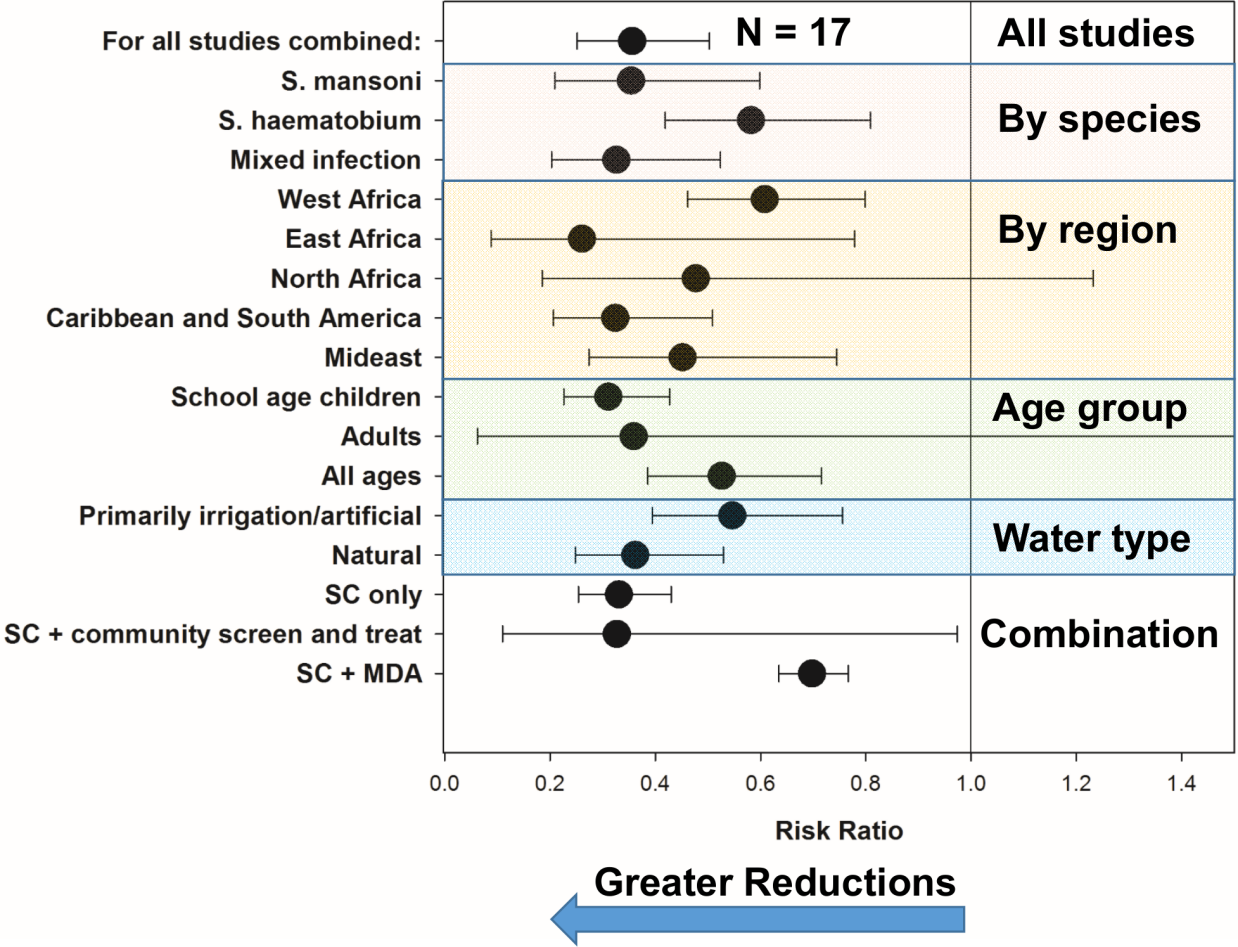
Risk ratios for interval *Schistosoma* infection incidence after implementation of mollusciciding programs. Black circles indicate risk ratios (RR) estimated by random effects meta-analysis for *Schistosoma* infection incidence after mollusciciding, as compared to pretreatment levels. Whisker bars indicate the 95% confidence interval for the RR estimates. Numeric details are provided in Supporting Information file S3 Table. Results are shown for all studies reporting human incidence data after implementation of control (top line, N = 17), and for study subgroup categories classified according to parasite species, geographic region, which age groups were included, the types of water bodies treated, and whether the program included snail control (SC) only, or mollusciciding combined with some form of drug treatment intervention, including targeted mass drug administration (MDA).

**Fig 7:**
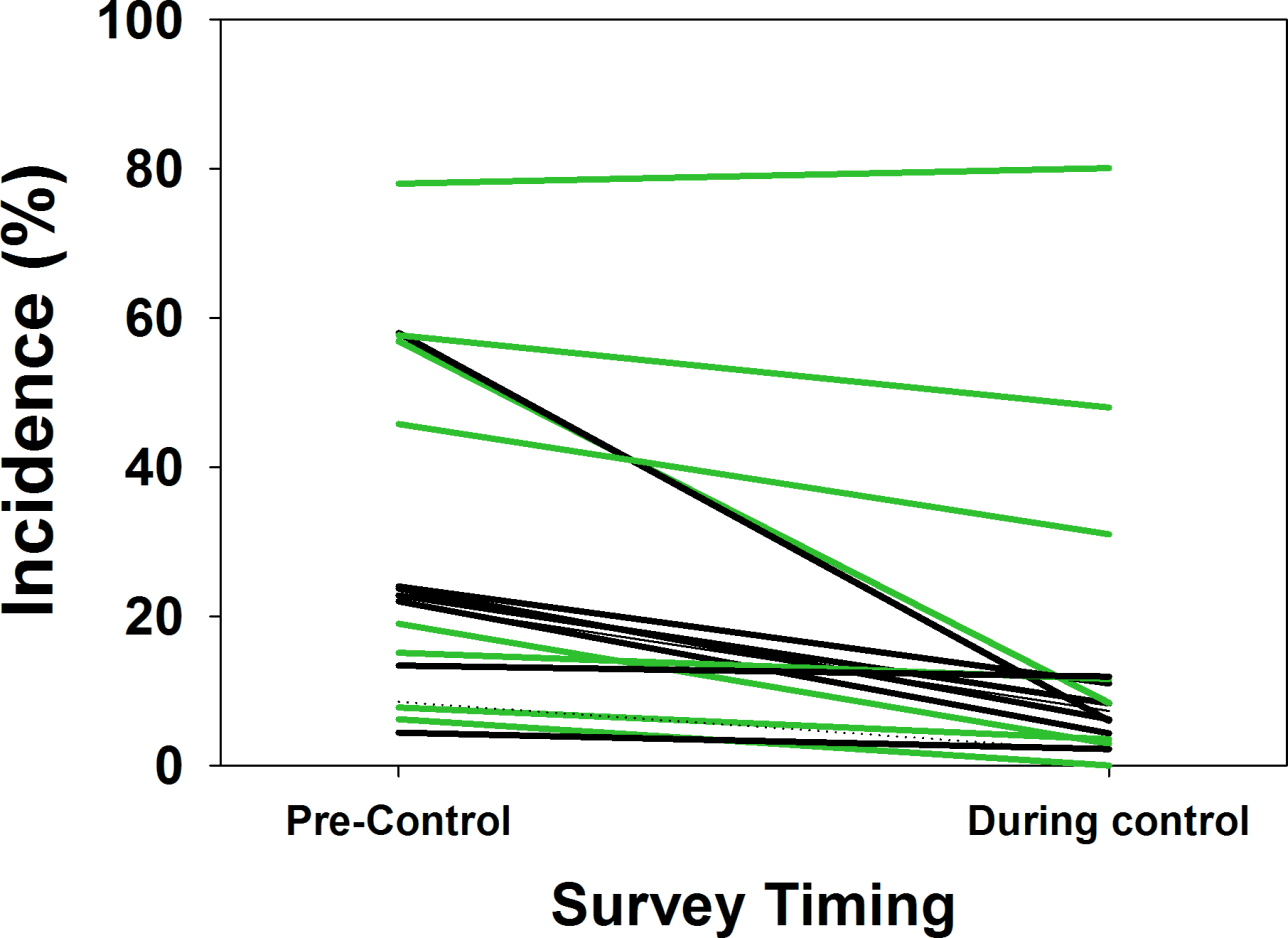
Change in local human incidence of *Schistosoma* infection during mollusciciding control projects. Individual lines represent the shift from pre-control incidence (left side) to incidence measured after implementation of chemical mollusciciding (right side) by schistosomiasis control programs. Black lines indicate programs that used niclosamide mollusciciding alone, green lines indicate programs that combined mollusciciding with anti-schistosomal drug treatments as part of control.

**Fig 8:**
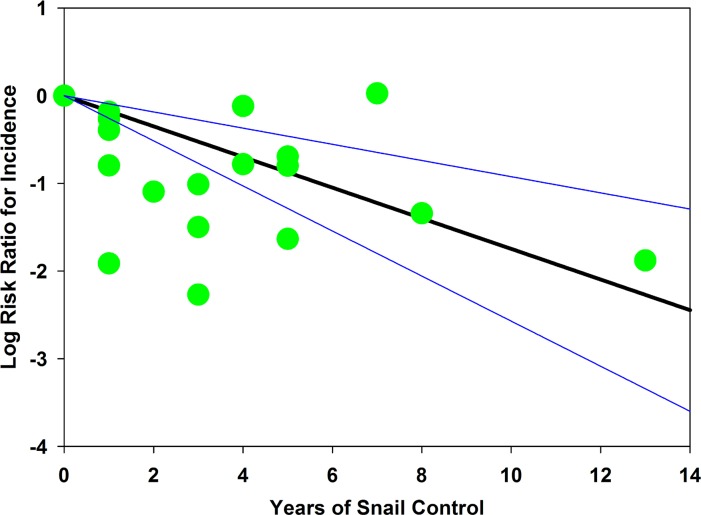
Meta-regression of *Schistosoma* infection incidence log-risk ratio according to the duration of mollusciciding control. Green circles indicate the point estimates for individual studies, graphed as the log risk ratio of infection after mollusciciding (y axis) against the reported duration of snail control (x axis). The dark line indicates the best-fit linear regression line for the combined studies, anchored at zero effect at time 0. The thin lines indicate the 95% confidence band for the regression.

Seventeen studies reported in 12 publications [28, 35, 38, 41, 50, 61, 67, 69, 71, 73, 76, 82] reported on the impact of mollusciciding programs on the incidence of new *Schistosoma* infections before and during control. As for prevalence, above, heterogeneity in incidence outcomes was high (I^2^ statistic = 93.2) among the reported studies. From our random effects meta-analysis of all 17 studies, the risk of new infection was estimated to be reduced by 64% (relative risk = 0.36, CI_95%_ 0.25, 0.50) in schistosomiasis control programs that included mollusciciding. The forest plot for studies reporting incidence outcomes is provided in Supporting Information file S2 Fig. Yearly incidence dropped from a median 22% (Range: 4% to 78%, IQR 14% to 54%) before intervention to a median 8% (Range: 2% to 80%, IQR 4% to 12%, *P* = 0.001 for the difference) during the course of these mollusciciding intervention programs. Fig 6 graphs summary estimates of the risk ratio for infection after molluscicide treatment intervention as compared to pre-treatment levels (numeric details are provided in Supporting Information file S3 Table). Of note, reduction of incidence was greater (i.e., RR was lower) for areas with natural water sources as compared to irrigation schemes (RR 0.36 vs. 0.55). There was also no apparent difference in incidence reduction effect when drug treatments were included in the control programs (RR for snail control alone was 0.33 vs. 0.32 for snail control plus community screening and drug treatment). Fig 7 shows the shift in pre- and post- incidence values for individual studies. Fig 8 graphs a meta-regression of the observed impact of mollusciciding on incidence (in terms of log-risk ratio) according to the duration of program implementation, indicating what appears to be an effect on incidence in most locations within 1–3 years.

### Impacts on incidence and prevalence in studies having concurrent control areas

As noted earlier, most included studies focused on one area using historical control data to assess impact. Nine studies reported in eight publications [22, 23, 27, 28, 38, 61, 71, 76] reported on concurrent infection outcomes in untreated areas near the molluscicide trial site. Table 1 summarizes the observed effects on prevalence and incidence of *Schistosoma* infection in the treated and untreated zones in each study. Of note, treatment and comparison zones had only one area unit each, and assignment was not randomized.

**Table 1:** Impact of mollusciciding on *Schistosoma* infection compared concurrently to transmission in untreated control areas.

			Prevalence		Incidence	
			Treated Zone	Untreated Zone	Treated Zone	Untreated Zone
Study first author, *[citation]*	Years treated	Country (*Species)* ^*a*^	*Before/after (% reduction)* ^b^		*Before/after (% reduction)* ^b^	
Barbosa [28]	1966–1974	Brazil (*Sm*)	0.73/0.31 (58%)	0.71/0.41 (42%)	0.24/0.06 (75%)	19%/7.5% (60%)
Farooq [71]	1962–1965	Egypt (*Sm*)	0.46/0.049 (89%)	0.26/0.29 (None)	0.085/0.019 (78%)	0.064/0.134 (None)
Farooq [71]	1962–1965	Egypt (*Sh*)	0.40/0.295 (26%)	0.49/0.58 (None)	0.23/0.083 (64%)	0.18/0.21 (None)
Ferguson^†^ [22]	1954–1967	Puerto Rico (*Sm*)	0.07/0.00 (100%)	0.11/0.012 (89%)	--/--	--/--
Jobin^†^ [23]	1953–1966	Puerto Rico (*Sm*)	0.16/0.00 (100%)	0.10/0.012 (88%)	--/--	--/--
Jordan [61]	1970–1975	St. Lucia (*Sm*)	0.34/0.23 (32%)	0.35/0.63 (None)	0.22/0.043 (80%)	0.22/0.20 (8%)
Kariuki [38]	1996–2001	Kenya (*Sm*)	0.66/0.22 (67%)	0.89/0.65 (27%)	--/--	--/--
Lyons [76]	1969–1971	Ghana (*Sh*)	0.38/0.29 (24%)	0.325/0.32 (1%)	0.22/0.073 (66%)	0.21/0.062 (70%)
Tamiem [27]	1980–1982	Sudan (*Sm*)	0.14/0.10 (29%)	0.51/0.61 (None)	--/--	--/--

^a^abbreviations: *Sm*, *Schistosoma mansoni* infection; *Sh*, *Schistosoma haematobium* infection

^b^Percent reduction, calculated as [(pre-control value- post-control value)/pre-control value]

^†^Linked studies sharing the same comparison area. Non-niclosamide molluscicides were used in treated areas.

In general, all molluscicide-treated areas saw greater declines in prevalence or incidence than untreated areas. Remarkably, though, prevalence fell significantly without molluscicide intervention (or population-based drug treatments) over a period 8 years in the Brazil study region [28] and over 13 years in Puerto Rican districts [22, 23], indicating interval changes in local risk for transmission that were unrelated to the snail control intervention. In the Ghana-2101 project, Lyons [76] observed a spontaneous drop in *S*. *haematobium* incidence over a 2 year period in an untreated comparison area, which was associated with an unexplained interval disappearance of local bulinid snails. Five study areas saw no change in prevalence in their untreated comparison areas but concurrent reductions in prevalence of 24% to 89% within snail control areas.

## Discussion

This systematic review and meta-analysis summarizes what has been a broad and lengthy experience with the use of mollusciciding for control of *S*. *mansoni* and *S*. *haematobium* transmission. Results of our analysis suggest that chemical-based snail control, particularly with the compound niclosamide, can effectively reduce local transmission of *Schistosoma* parasites when delivered at regular interval and under skilled supervision [17]. Direct treatment effects on snails were difficult to summarize, because of the many differences in sampling and reporting used in the included studies. However, where snail reductions were quantified, most programs saw very significant reductions or complete disappearance of local *Schistosoma* host snails during program implementation. Earlier studies tended to favor broad, intensive mollusciciding in an attempt to eliminate intermediate host *Biomphalaria* or *Bulinus* spp. snails. With such approaches, and particularly where water flow could be controlled in irrigation systems and transmission was more seasonal, treatment intervals could be extended to 6–12 months [8, 79, 83]. Later studies, often dealing with natural water bodies and more focal human water contact, tended to favor more frequent focal administration of mollusciciding [13, 41, 43, 62, 66, 84], allowing snails to persist elsewhere outside the main human water contact zones. While not fully explored, several preliminary studies suggested that slow-release strips or pellet formulations [85, 86] or delayed-release molluscicide capsules [87] might better focus the impact of chemical molluscicide and extend its duration of impact, with concomitant cost-savings due to a reduced need for frequent delivery.

Given the cumulative experience of the programs summarized here, it becomes clear that focality of snail habitats, combined with overlap into human water contact zones, represent factors in successful *Schistosoma* transmission. Not all waterbodies within a control area are suitable for host snails [88], but human movement among water contact sites can strongly facilitate regional persistence of transmission [89, 90]. For these reasons, implementation of focal snail control requires an adequate surveillance component to have an accurate working knowledge of local snail habitat, of human water contact zones, and of the seasonal factors affecting the abundance of snails and the likelihood of transmission [66, 73, 91].

As Shiff [92] points out, the ultimate value of a mollusciciding campaign is measured by its impact on human infection. Where snail control was used alone, early reductions in human incidence and later reductions in human *Schistosoma* infection prevalence could usually be obtained. When snail control was combined with population screening and selective or mass drug therapy, prevalence was reduced more quickly and incidence diminished. However, transmission was frequently not eliminated. When most successful, programs involving snail control achieved 85–100% reductions in local prevalence of *Schistosoma* infections [8, 22, 23, 68–71, 75, 78]. However, some control programs appeared to have only minimal impact on local prevalence [39, 66, 73, 77, 81]. While there were some apparent differences in effects by region and by parasite species, the snail species named in the included studies were too diverse to draw meaningful comparisons for prevalence or incidence outcomes stratified at the intermediate snail host species level.

Authors cited the fact that established control is vulnerable to resurgence of snail populations from local refugia [93–95]. The homing characteristics of miracidia for host snails, combined with the homing of cercariae towards human skin and the high degree of asexual multiplication within infected snails, strongly favor persistence of transmission [70]. Given the presence of untreated human individuals within the control area, whether from refusal of drug therapy or in-migration from (or temporary travel to) areas not under *Schistosoma* transmission control [64, 70, 76, 77, 96], new infections are likely to continue to occur. Habitat changes, growth in human populations, and breakdowns in program performance are other factors that can contribute to limit the impact of snail control programs—whereas Egypt did very well with mollusciciding campaigns in the 1960s [71], by the 1980s their programs were having limited effectiveness [66, 73]. An independent on site WHO review performed in 1985 identified gaps in communication between snail control teams and health personnel, errors in selection of snail sampling sites, inefficiencies in snail testing, and an over-reliance on infrequent area-wide mollusciciding (as opposed to more frequent focal mollusciciding) as contributing causes to poor performance in that era [66].

The evidence summarized in this meta-analysis appears, in general, to favor mollusciciding as an effective method to reduce *Schistosoma* infections over time, with an additive effect on prevalence where population-based drug control is also given. However, the quality of the reported evidence is limited. The studies included in the analysis were non-randomized interventional trials, often with only historical data used for comparison in assessing the magnitude of snail control outcomes [26]. Where concurrent comparison areas were used, transmission was inconsistent in some areas, suggesting that secular trends or temporal fluctuations were occurring, which means that there is risk of over- or under-estimating the impact of mollusciciding in studies that use only historical controls. As such, and in view of the variability of ecology in *Schistosoma* transmission settings, the formal scientific evidence for a ‘generalizable’ consistent effect of snail control can be considered only minimally strong at this time. Other limitations in performing our analysis stem from study-to-study differences in snail control implementation, measures of snail impact, monitoring of human population outcomes, and duration of control.

For the reader considering implementation of a snail control program based on niclosamide mollusciciding, the 1983 monograph by Andrews, et al. [46] provides extensive information on the chemistry, biology, and toxicology of niclosamide, as well as its effects on non-targeted plant and animal species. Niclosamide’s environmental impacts have been more recently reviewed by Dawson [97], who concluded that there is minimal risk to humans and the environment, provided its application is appropriately dose-limited, informed, and supervised. It is important to be aware, however, that niclosamide is harmful to fish, amphibia, certain insect larvae, and in higher doses, to aquatic vegetation [46, 97, 98]. Because niclosamide quickly decays over 24 hours, animals that can rapidly move away from an area of application may return in a matter of days [62]. In aggregate, it appears that metered and very focal niclosamide administration at human water contact sites has the potential to provide the greatest impact on *Schistosoma* transmission with the least impact on local ecosystems.

Overall, the impacts reported in the included studies predominantly lean toward a positive effect of mollusciciding in reducing *Schistosoma* transmission, with longer duration of control leading to a greater impact. These findings hold promise that the benefits of mollusciciding could be further defined in modern, well-designed comparison trials. A randomized comparison trial of mass drug administration ± snail control is in now progress in Zanzibar as part of a program that is attempting local elimination of *S*. *haematobium* [99]. Additional mollusciciding trials for *S*. *mansoni* control and elimination are under development. We look forward to the results of these efforts, which are expected to provide valuable evidence to further inform *Schistosoma* control policy. Based on past experience, regular focal mollusciciding is likely to contribute significantly to the move toward elimination of schistosomiasis in high risk areas.

## Supporting Information

S1 Dataset Data used for meta-analysis of prevalence outcomes. (XLSX)

S2 Dataset Data used for meta-analysis of incidence outcomes. (XLSX)

S1 Fig Forest plots for meta-analysis of prevalence effects of mollusciciding programs. (PPTX)

S2 Fig Forest plots for meta-analysis of incidence effects of mollusciciding programs. (PPTX)

S3 Fig Forest plot of mollusciciding impact on area prevalence, sorted by starting prevalence. (PDF)

S1 Table Impact of molluscicide treatment for reduction of *Schistosoma* infection prevalence: Sub-group analysis. Estimated effects of molluscicide intervention by parasite species, region, age-groups monitored, water habitat, and the inclusion of different strategies of drug treatment. These data are presented graphically in Fig 4 of the paper.
(DOCX)

S2 Table Characteristics of six studies showing only limited impact of mollusciciding campaigns on local prevalence. (XLSX)

S3 Table Impact of molluscicide treatment on incidence of *Schistosoma* infections: Sub-group analysis. Estimated effects of molluscicide intervention by parasite species, region, age-groups monitored, water habitat, and the inclusion of different strategies of drug treatment. These data are presented graphically in Fig 6 of the paper.
(DOCX)

S1 File PRISMA checklist. (DOC)

S2 File Study protocol. Prospero number CRD42013006869
(PDF)

S3 File Listing of included studies. Papers abstracted for data on the extent and duration of snail number reduction and/or the impact of mollusciciding on local human prevalence and incidence of *Schistosoma* infection.
(DOCX)

S4 File Listing of excluded studies. Papers reviewed but not included in the qualitative or quantitative analyses.
(DOCX)

S5 File Funnel plot and Egger’s Regression Analysis. Graphical and regression analysis for potential publication bias of studies included in the meta-analysis, measuring, respectively, the impact of mollusciciding on local human prevalence (page 1) and incidence (page 2) of *Schistosoma* infection.
(PDF)
