# Supplementary figures and images for: Systematic Review and Meta-analysis of the Impact of Chemical-Based Mollusciciding for Control of Schistosoma mansoni and S. haematobium Transmission
Source: PLoS Negl Trop Dis. 2015 Dec 28;9(12):e0004290. doi: 10.1371/journal.pntd.0004290 (PMC4692485; doi:10.1371/journal.pntd.0004290)

## Slide 1
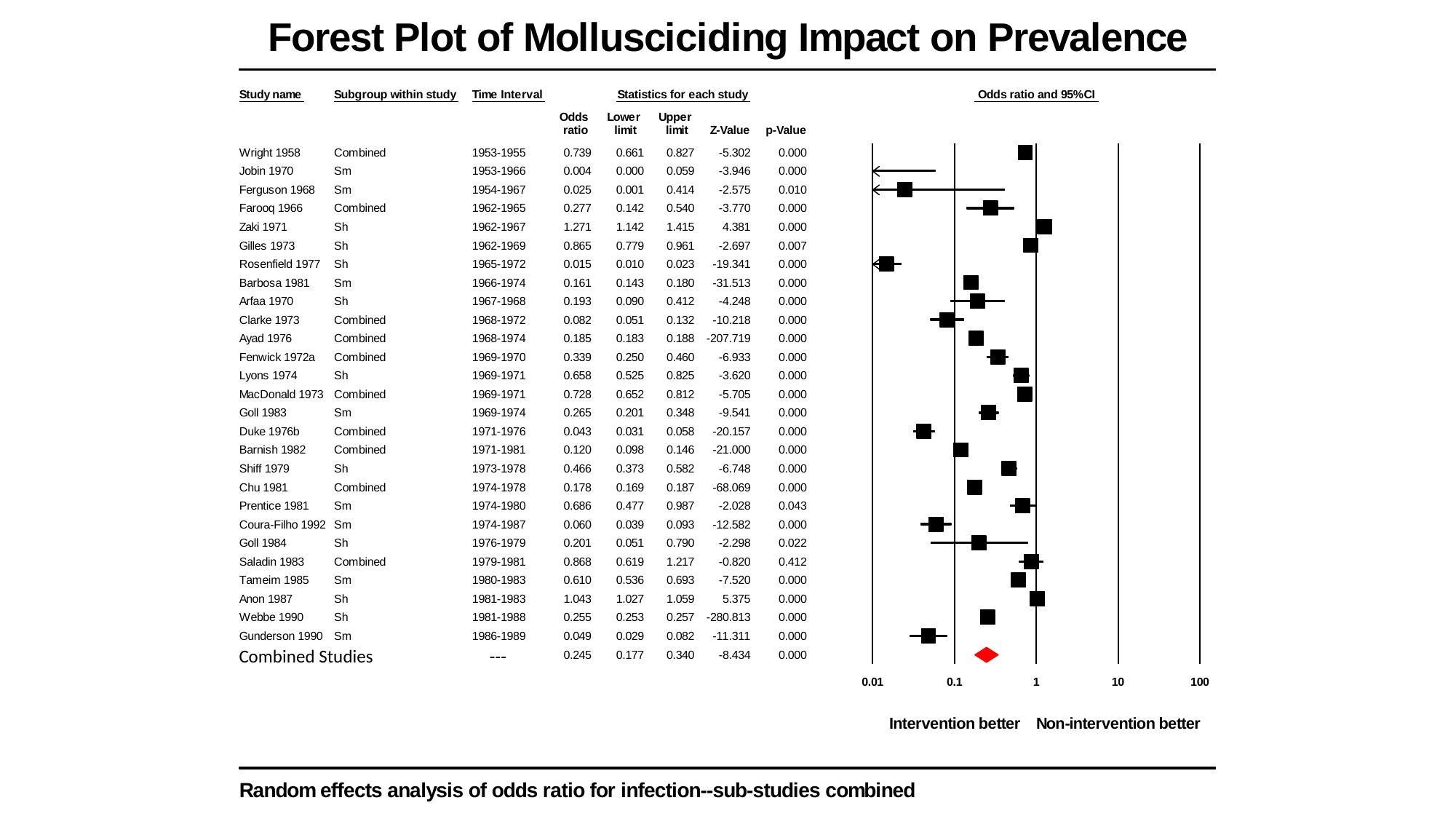

Combined Studies ---

Supplement: S1 Fig — (PPTX) [file pntd.0004290.s003.pptx]

## Slide 1
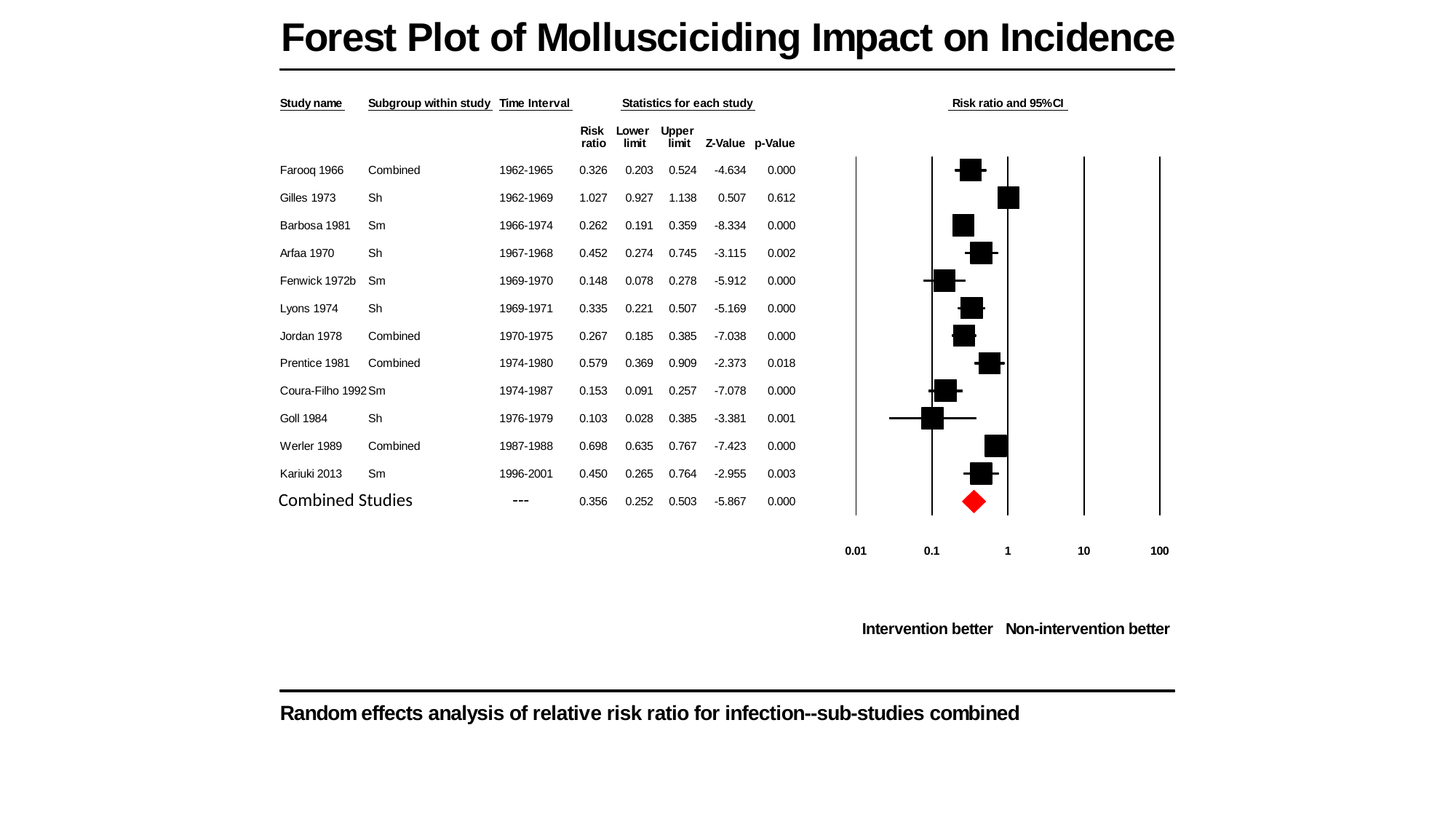

Combined Studies ---

Supplement: S2 Fig — (PPTX) [file pntd.0004290.s004.pptx]
