# Supplementary material for: Systematic Review and Meta-analysis of the Impact of Chemical-Based Mollusciciding for Control of Schistosoma mansoni and S. haematobium Transmission
Source: PLoS Negl Trop Dis. 2015 Dec 28;9(12):e0004290. doi: 10.1371/journal.pntd.0004290 (PMC4692485; doi:10.1371/journal.pntd.0004290)
Supplement: S3 Fig — (PDF) [file pntd.0004290.s005.pdf]

Odds Ratio of Infection Prevalence after Introduction of Mollusciciding

Highest starting prevalence

Study name	Subgroup within study	Time Interval	Odds ratio	Lower limit	Upper limit	Z-Value	p-Value
			**Statistics for each study**				
Duke 1976b High	Sh	1971-1976	0.035	0.023	0.052	-16.363	0.000
Rosenfield 1977	Sh	1965-1972	0.015	0.010	0.023	-19.341	0.000
Chu 1981 High	Sh	1974-1978	0.197	0.182	0.214	-39.984	0.000
Duke 1976b Low	Sh	1971-1976	0.036	0.020	0.066	-10.882	0.000
Barbosa 1981	Sm	1966-1974	0.161	0.143	0.180	-31.513	0.000
Shiff 1979	Sh	1973-1978	0.466	0.373	0.582	-6.748	0.000
Gunderson 1990	Sm	1986-1989	0.049	0.029	0.082	-11.311	0.000
Chu 1981 Low	Sh	1974-1978	0.119	0.111	0.129	-57.113	0.000
Goll 1983	Sm	1969-1974	0.265	0.201	0.348	-9.541	0.000
Saladin 1983	Combined	1979-1981	0.868	0.619	1.217	-0.820	0.412
Clarke 1973	Combined	1968-1972	0.082	0.051	0.132	-10.218	0.000
Fenwick 1972a SAC	Sm	1969-1970	0.186	0.063	0.551	-3.036	0.002
MacDonald 1973	Combined	1969-1971	0.728	0.652	0.812	-5.705	0.000
Prentice 1981	Sm	1974-1976	0.686	0.477	0.987	-2.028	0.043
Ayad 1976	Combined	1968-1974	0.185	0.183	0.188	-207.719	0.000
Farooq 1966	Combined	1962-1965	0.277	0.142	0.540	-3.770	0.000
Barnish 1982 High	Sm	1971-1981	0.067	0.052	0.086	-21.166	0.000
Fenwick 1972a Adults	Sm	1969-1970	0.359	0.261	0.494	-6.293	0.000
Coura-Filho 1992	Sm	1974-1987	0.060	0.039	0.093	-12.582	0.000
Wright 1958	Combined	1953-1955	0.739	0.661	0.827	-5.302	0.000
Pesigan 1961	Sj	1957-1959	0.739	0.636	0.860	-3.922	0.000
Lyons 1974	Sh	1969-1971	0.658	0.525	0.825	-3.620	0.000
Zaki 1971	Sh	1962-1967	1.271	1.142	1.415	4.381	0.000
Gilles 1973	Sh	1962-1969	0.865	0.779	0.961	-2.697	0.007
Anon 1987	Sh	1981-1983	1.043	1.027	1.059	5.375	0.000
Webbe 1990	Sh	1981-1988	0.255	0.253	0.257	-280.813	0.000
Goll 1984	Sh	1976-1979	0.201	0.051	0.790	-2.298	0.022
Jobin 1970	Sm	1953-1966	0.004	0.000	0.059	-3.946	0.000
Tameim 1985	Sm	1980-1983	0.610	0.536	0.693	-7.520	0.000
Barnish 1982 Low	Sm	1971-1981	0.262	0.188	0.365	-7.916	0.000
Arfaa 1970	Sh	1967-1968	0.193	0.090	0.412	-4.248	0.000
Ferguson 1968	Sm	1954-1967	0.025	0.001	0.414	-2.575	0.010
			0.229	0.169	0.309	-9.571	0.000

Lowest starting prevalence

Odds ratio and 95%CI

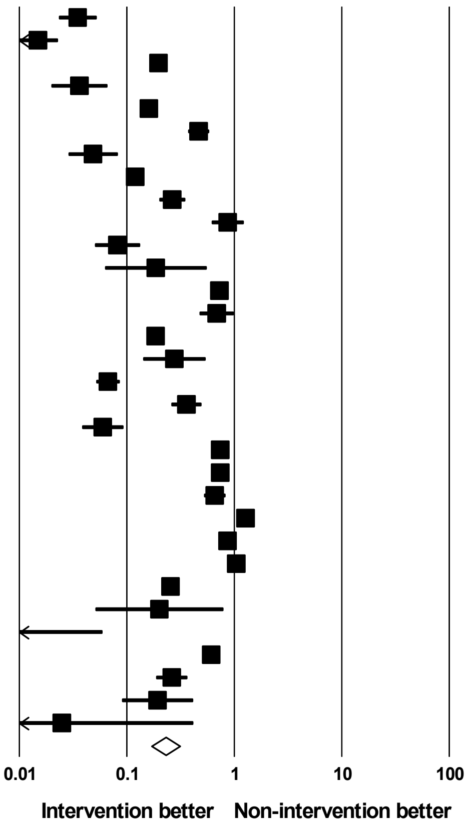

Random effects analysis by starting prevalence
