# Supplementary material for: Systematic Review and Meta-analysis of the Impact of Chemical-Based Mollusciciding for Control of Schistosoma mansoni and S. haematobium Transmission
Source: PLoS Negl Trop Dis. 2015 Dec 28;9(12):e0004290. doi: 10.1371/journal.pntd.0004290 (PMC4692485; doi:10.1371/journal.pntd.0004290)
Supplement: S1 Table — Estimated effects of molluscicide intervention by parasite species, region, age-groups monitored, water habitat, and the inclusion of different strategies of drug treatment. These data are presented graphically in Fig 4 of the paper. (DOCX) [file pntd.0004290.s006.docx]

**Table S1. Impact of molluscicide treatment for reduction of *Schistosoma* infection prevalence: sub-group analysis.** Estimated effects of molluscicide intervention by parasite species, region, age-groups monitored, water habitat, and the inclusion of different strategies of drug treatment. These data are presented graphically in Figure 4 of the paper.

| **Subgroup** | **Number in subgroup** | **Odds Ratio of having infection following introduction of mollusciciding** | **CI_95%_ for Group Odds Ratio** |
| --- | --- | --- | --- |
| **For all studies combined:** | 35 | 0.229 | 0.169, 0.309 |
| **By parasite species:** |  |  |  |
| *S. mansoni* | 16 | 0.184 | 0.109, 0.311 |
| *S. haematobium* | 17 | 0.241 | 0.150, 0.387 |
| Mixed infection | 2 | 0.397 | 0.088, 1.804 |
| **By region:** |  |  |  |
| West Africa | 8 | 0.247 | 0.099, 0.619 |
| East Africa | 4 | 0.235 | 0.106, 0.522 |
| North Africa | 10 | 0.532 | 0.285, 0.993 |
| Southern Africa | 5 | 0.315 | 0.136, 0.733 |
| Caribbean and South America | 7 | 0.133 | 0.071, 0.247 |
| Mideast | 1 | 0.193 | 0.090, 0.412 |
| **By patient age group monitored:** |  |  |  |
| School age children | 14 | 0.365 | 0.172, 0.774 |
| All ages | 21 | 0.193 | 0.144, 0.258 |
| **By water habitat:** |  |  |  |
| Primarily irrigation/artificial | 13 | 0.328 | 0.208, 0.515 |
| Natural | 15 | 0.194 | 0.115, 0.327 |
| **By single *vs.* combined intervention:** |  |  |  |
| Snail control only | 10 | 0.470 | 0.276, 0.800 |
| Snail control plus community screening and treatment | 19 | 0.162 | 0.116, 0.225 |
| Snail control plus school based screening and treatment | 3 | 0.440 | 0.081, 2.389 |
| Snail control plus mass drug administration | 3 | 0.207 | 0.012, 3.491 |
