# Supplementary material for: Systematic Review and Meta-analysis of the Impact of Chemical-Based Mollusciciding for Control of Schistosoma mansoni and S. haematobium Transmission
Source: PLoS Negl Trop Dis. 2015 Dec 28;9(12):e0004290. doi: 10.1371/journal.pntd.0004290 (PMC4692485; doi:10.1371/journal.pntd.0004290)
Supplement: S3 Table — Estimated effects of molluscicide intervention by parasite species, region, age-groups monitored, water habitat, and the inclusion of different strategies of drug treatment. These data are presented graphically in Fig 6 of the paper. (DOCX) [file pntd.0004290.s008.docx]

**S3 Table. Impact of molluscicide treatment on incidence of *Schistosoma* infections: sub-group analysis.** Estimated effects of molluscicide intervention by parasite species, region, age-groups monitored, water habitat, and the inclusion of different strategies of drug treatment. These data are presented graphically in Figure 6 of the paper.

| **Subgroup** | **Number in subgroup** | **Risk Ratio of new infection following introduction of mollusciciding** | **CI_95%_ for Group Risk Ratio** |
| --- | --- | --- | --- |
| **For all studies combined:** | 17 | 0.356 | 0.252, 0.503 |
| **By parasite species:** |  |  |  |
| *S. mansoni* | 9 | 0.354 | 0.209, 0.598 |
| *S. haematobium* | 7 | 0.582 | 0.419, 0.809 |
| Mixed infection | 1 | 0.326 | 0.203, 0.524 |
| **By region:** |  |  |  |
| West Africa | 5 | 0.608 | 0.462, 0.799 |
| East Africa | 2 | 0.261 | 0.088, 0.779 |
| North Africa | 3 | 0.477 | 0.185, 1.232 |
| Caribbean and South America | 6 | 0.324 | 0.207, 0.509 |
| Mideast | 1 | 0.452 | 0.274, 0.745 |
| **By patient age group monitored:** |  |  |  |
| School age children | 7 | 0.311 | 0.226, 0.427 |
| Adults | 2 | 0.359 | 0.062, 2.086 |
| All ages | 8 | 0.526 | 0.386, 0.716 |
| **By water habitat:** |  |  |  |
| Primarily irrigation/artificial | 6 | 0.546 | 0.394, 0.756 |
| Natural | 11 | 0.362 | 0.248, 0.529 |
| **By single vs. combined intervention:** |  |  |  |
| Snail control only | 10 | 0.331 | 0.254, 0.430 |
| Snail control plus community screening and treatment | 4 | 0.327 | 0.110, 0.974 |
| Snail control plus mass drug administration | 3 | 0.698 | 0.635, 0.767 |
