# Supplementary material for: Systematic Review and Meta-analysis of the Impact of Chemical-Based Mollusciciding for Control of Schistosoma mansoni and S. haematobium Transmission
Source: PLoS Negl Trop Dis. 2015 Dec 28;9(12):e0004290. doi: 10.1371/journal.pntd.0004290 (PMC4692485; doi:10.1371/journal.pntd.0004290)
Supplement: S2 File — Prospero number CRD42013006869 (PDF) [file pntd.0004290.s010.pdf]

PROSPERO International prospective register of systematic reviews

Review title and timescale

1 Review title
Give the working title of the review. This must be in English. Ideally it should state succinctly the interventions or exposures being reviewed and the associated health or social problem being addressed in the review.
Snail control by niclosamide mollusciciding for prevention of schistosomiasis

2 Original language title
For reviews in languages other than English, this field should be used to enter the title in the language of the review. This will be displayed together with the English language title.

3 Anticipated or actual start date
Give the date when the systematic review commenced, or is expected to commence.
16/12/2013

4 Anticipated completion date
Give the date by which the review is expected to be completed.
31/03/2015

5 Stage of review at time of this submission
Indicate the stage of progress of the review by ticking the relevant boxes. Reviews that have progressed beyond the point of completing data extraction at the time of initial registration are not eligible for inclusion in PROSPERO. This field should be updated when any amendments are made to a published record.

The review has not yet started ×

Review stage	Started	Completed
Preliminary searches	Yes	Yes
Piloting of the study selection process	Yes	Yes
Formal screening of search results against eligibility criteria	Yes	Yes
Data extraction	Yes	Yes
Risk of bias (quality) assessment	Yes	Yes
Data analysis	Yes	Yes

Provide any other relevant information about the stage of the review here.
funded

Review team details

6 Named contact
The named contact acts as the guarantor for the accuracy of the information presented in the register record.
Charles King

7 Named contact email
Enter the electronic mail address of the named contact.
chk@cwru.edu

8 Named contact address
Enter the full postal address for the named contact.
Center for Global Health and Diseases Case Western Reserve University School of Medicine 2109 Adelbert Road, BRB 422 Cleveland OH 44106 USA

9 Named contact phone number
Enter the telephone number for the named contact, including international dialing code.
1-216-368-3667

10 Organisational affiliation of the review
Full title of the organisational affiliations for this review, and website address if available. This field may be completed

as 'None' if the review is not affiliated to any organisation.
Case Western Reserve University

Website address:
http://www.case.edu/orgs/cghd/

11 Review team members and their organisational affiliations
Give the title, first name and last name of all members of the team working directly on the review. Give the organisational affiliations of each member of the review team.

Title	First name	Last name	Affiliation
Dr	Charles	King	Center for Global Health, CWRU
Mr	David	Bertsch	Center for Global Health, CWRU
Ms	Laura	Sutherland	Ohio State University

12 Funding sources/sponsors
Give details of the individuals, organizations, groups or other legal entities who take responsibility for initiating, managing, sponsoring and/or financing the review. Any unique identification numbers assigned to the review by the individuals or bodies listed should be included.
Schistosomiasis Consortium for Operational Research and Evalaution

13 Conflicts of interest
List any conditions that could lead to actual or perceived undue influence on judgements concerning the main topic investigated in the review.
Are there any actual or potential conflicts of interest?
None known

14 Collaborators
Give the name, affiliation and role of any individuals or organisations who are working on the review but who are not listed as review team members.

Title	First name	Last name	Organisation details

Review methods

15 Review question(s)
State the question(s) to be addressed / review objectives. Please complete a separate box for each question.
Does chemical mollusciciding effectively reduce snail numbers in a manner to prevent reinfection or new infection in at risk human populations?

16 Searches
Give details of the sources to be searched, and any restrictions (e.g. language or publication period). The full search strategy is not required, but may be supplied as a link or attachment.
Electronic searches Computer database search using double combinations of the terms 'Molluscicide'; 'snail control/prevention'; 'Biomphalaria'; 'Bulinus'; 'field [trial]' "schistosomiasis/prevention and control" "transmission", 'niclosamide" Secondary report finding will be done by scanning PubMed 'similar articles' feature, and by using the Google- and PubMed-generated listings of papers that cite our already-included papers, i.e., those that we found to contain well-conducted snail control intervention trails. No limits on language or dates. Databases queried: PubMed, Google Scholar, Scielo, African Journals Online Searching other resources Hand searches of WHO technical reports, and of archived files at CWRU and SCORE.

17 URL to search strategy
If you have one, give the link to your search strategy here. Alternatively you can e-mail this to PROSPERO and we will store and link to it.

I give permission for this file to be made publicly available
Yes

18 Condition or domain being studied

Give a short description of the disease, condition or healthcare domain being studied. This could include health and wellbeing outcomes.
Schistosomiasis, the chronic disease caused by Schistosoma spp. parasite infections of humans, is a preventable illness that, if left under-treated, is associated with long-term under-nutrition, anemia, organ fibrosis and disabling patient symptoms. Optimal disease prevention can occur only when parasite infection or reinfection can be effectively blocked. Alone, Preventive Chemotherapy (PCT) via mass drug administration has not been very successful in limiting transmission in high-risk areas.The WHO's new focus on 'transmission control, wherever possible' means it is appropriate to re-examine the efficacy of intermediate-host snail control for prevention of human-to-snail-to-human parasite transmission. Reduction in infected snail numbers at the places where humans come into contact with freshwater could substantially reduce each patient's frequency of exposure to infecting parasite larvae (cercariae), and, hence, reduce the frequency of reinfection.

19 Participants/population
Give summary criteria for the participants or populations being studied by the review. The preferred format includes details of both inclusion and exclusion criteria.
Field trials of niclosamide application to water bodies in Schistosoma endemic areas for experimental or programmatic control of snail intermediate hosts for S. mansoni = Biomphalaria spp. and for S. haematobium = Bulinus spp.

20 Intervention(s), exposure(s)
Give full and clear descriptions of the nature of the interventions or the exposures to be reviewed
The trial should include periodic application of niclosamide compounds to transmission water contact sites or experimental locations. Data should include snail species, treatment dose, frequency, habitat (static vs. flowing water), region, and season of application. Information about local human prevalence and incidence of Schistosoma infection, before and after intervention, will be evalauated as a secondary outcome.

21 Comparator(s)/control
Where relevant, give details of the alternatives against which the main subject/topic of the review will be compared (e.g. another intervention or a non-exposed control group).
Snail survival in control, untreated locations during the same period as the treatment trial.

22 Types of study to be included initially
Give details of the study designs to be included in the review. If there are no restrictions on the types of study design eligible for inclusion, this should be stated.
No restrictions in terms of language or year. Because niclosamide performance has been recently reviewed for China (Yang, et al., Parasites & Vectors, 2011) Chinese studies will be excluded from the present meta-analysis, whcih will focus on Africa, the Mideast, and S. America.

23 Context
Give summary details of the setting and other relevant characteristics which help define the inclusion or exclusion criteria.
Theoretical modelling (MacDonad 1965) suggests that >90% reduction in snail numbers, in conjunction with population drug treatment, has the potential to extinguish Schistosoma populations from local ecosystems. Chemical molluscicides, including niclosamide (Bayluscide, Bayer 73) were used extensively in the 1960s and 1970s for schistosomiasis control in Africa, but these have not had much use in the last 40-50 years. It is important to systematically review the efficacy of such treatments in snail suppression, and infection prevention, so that approaches to integrated Schistosoma control can be modeled and costed in conjunction with plans for targeted elimination in high-risk locations.

24 Primary outcome(s)
Give the most important outcomes.
Snail mortality after treatment.

Give information on timing and effect measures, as appropriate.
As dependent on snail species, treatment dose, frequency, habitat (static vs. flowing water), region, and seasonality.

25 Secondary outcomes
List any additional outcomes that will be addressed. If there are no secondary outcomes enter None.
Post-treatment snail abundance in treated locations over time. Rate of human infection or reinfection in treated communities. Studies with comparison ro concurrent untreated areas preferred.

Give information on timing and effect measures, as appropriate.

26 Data extraction, (selection and coding)
Give the procedure for selecting studies for the review and extracting data, including the number of researchers involved and how discrepancies will be resolved. List the data to be extracted.
Review of titles and abstracts by two trained reviewers, searching for data content meeting study requirements. Abstraction by two reviewers of treatment and location parameters, along with snail mortality at different time points following administration.

27 Risk of bias (quality) assessment
State whether and how risk of bias will be assessed, how the quality of individual studies will be assessed, and whether and how this will influence the planned synthesis.
As possible. Research performed in the pre-1980s era often did not report quality-related details in publications. Many of the researchers involved are now deceased or retired, without access to primary data.

28 Strategy for data synthesis
Give the planned general approach to be used, for example whether the data to be used will be aggregate or at the level of individual participants, and whether a quantitative or narrative (descriptive) synthesis is planned. Where appropriate a brief outline of analytic approach should be given.
Aggregate data from each study on percent snail numbers killed/total snails at treated sites will be used in meta-analysis for summary estimation of treatment efficacy, as dependent on dose, duration, frequency, species, water habitat and location

29 Analysis of subgroups or subsets
Give any planned exploration of subgroups or subsets within the review. ‘None planned’ is a valid response if no subgroup analyses are planned.
Outcomes will be tested for heterogeneity of results by chi-squared and i-squared statistics, and subgroup assessment used to explore causes for observed heterogeneity.

Review general information

30 Type of review
Select the type of review from the drop down list.
Intervention

31 Language
Select the language(s) in which the review is being written and will be made available, from the drop down list. Use the control key to select more than one language.
English

Will a summary/abstract be made available in English?
Yes

32 Country
Select the country in which the review is being carried out from the drop down list. For multi-national collaborations select all the countries involved. Use the control key to select more than one country.
United States of America

33 Other registration details
List places where the systematic review title or protocol is registered (such as with he Campbell Collaboration, or The Joanna Briggs Institute). The name of the organisation and any unique identification number assigned to the review by that organization should be included.

34 Reference and/or URL for published protocol
Give the citation for the published protocol, if there is one.
Give the link to the published protocol, if there is one. This may be to an external site or to a protocol deposited with CRD in pdf format.

I give permission for this file to be made publicly available
Yes

35 Dissemination plans
Give brief details of plans for communicating essential messages from the review to the appropriate audiences.
Presentations to SCORE annual meeting and to the American Society of Tropical Medicine National meeting 2014. Later publication in open access journal and archiving of data and background materials at the SCORE website.

Do you intend to publish the review on completion?
Yes

36 Keywords
Give words or phrases that best describe the review. (One word per box, create a new box for each term)
schistosomiasis

schistosoma

molluscicide

snail control

Biomphalaria

Bulinus

niclosamide

Bayluscide

Bayer 73

transmission

prevention

control

37 Details of any existing review of the same topic by the same authors
Give details of earlier versions of the systematic review if an update of an existing review is being registered, including full bibliographic reference if possible.
None

38 Current review status
Review status should be updated when the review is completed and when it is published.
Completed but not published

01/07/2015

39 Any additional information
Provide any further information the review team consider relevant to the registration of the review.

40 Details of final report/publication(s)
This field should be left empty until details of the completed review are available.
Give the full citation for the final report or publication of the systematic review.
Give the URL where available.
