# Supplementary material for: Systematic Review and Meta-analysis of the Impact of Chemical-Based Mollusciciding for Control of Schistosoma mansoni and S. haematobium Transmission
Source: PLoS Negl Trop Dis. 2015 Dec 28;9(12):e0004290. doi: 10.1371/journal.pntd.0004290 (PMC4692485; doi:10.1371/journal.pntd.0004290)
Supplement: S3 File — Papers abstracted for data on the extent and duration of snail number reduction and/or the impact of mollusciciding on local human prevalence and incidence of Schistosoma infection. (DOCX) [file pntd.0004290.s011.docx]

Papers abstracted for data on the extent and duration of snail number reduction and/or the impact of mollusciciding on local prevalence and incidence.

1. Anonymous. Report of an independent evaluation mission on the national control programme in Egypt-1985. Trans R Soc Trop Med Hyg. 1987;81(supplement):1-57.

2. Amin MA, Fenwick A. The development of an annual regimen for blanket snail control on the Gezira Irrigated Area of the Sudan. Ann Trop Med Parasitol. 1977;71(2):205-12. PubMed PMID: 869609.

3. Arfaa F, Farahmandian I, Sahba GH, Bijan H. Progress towards the control of bilharziasis in Iran. Trans R Soc Trop Med Hyg. 1970;64(6):912-7. PubMed PMID: 5495641.

4. Ayad N. Snail control and some significant control projects, review. Egyptian Journal of Bilharziasis. 1976;3(2):129-55. PubMed PMID: 1032289.

5. Barbosa FS, Costa DP. A long-term schistosomiasis control project with molluscicide in a rural area of Brazil. Ann Trop Med Parasitol. 1981;75(1):41-52. PubMed PMID: 7271354.

6. Barnish G, Jordan P, Bartholomew RK, Grist E. Routine focal mollusciciding after chemotherapy to control *Schistosoma mansoni* in Cul de Sac valley, Saint Lucia. Trans R Soc Trop Med Hyg. 1982;76(5):602-9. PubMed PMID: 7179412.

7. Berrios-Duran LA, Ritchie LS, Wessel HB. Field screening tests on molluscicides against *Biomphalaria glabrata* in flowing water. Bull World Health Organ. 1968;39(2):316-20. PubMed PMID: 5303414; PubMed Central PMCID: PMC2554541.

8. Choudhry AW. Seven years of snail control at MWEA Irrigation Settlement, Kenya: results and costs. East Afr Med J. 1974;51(8):600-9. PubMed PMID: 4472937.

9. Chu KY. Trials of ecological and chemical measures for the control of *Schistosoma haematobium* transmission in a Volta Lake village. Bull World Health Organ. 1978;56(2):313-22. PubMed PMID: 307458; PubMed Central PMCID: PMC2395562.

10. Chu KY, Klumpp RK, Kofi DY. Results of three years of cercarial transmission control in the Volta Lake. Bull World Health Organ. 1981;59(4):549-54. PubMed PMID: 6976224; PubMed Central PMCID: PMC2396095.

11. Clarke VV, Blair DM, Weber MC. The Mayfield experiment: an attempt to eliminate bilharziasis from a small community by repeated treatment of the infected people combined with intensive snail control. Cent Afr J Med. 1973:14-22. PubMed PMID: 4795848.

12. Coura Filho P, Rocha RS, de Lima ECMF, Katz N. A municipal level approach to the management of schistosomiasis control in Peri-Peri, MG, Brazil. Rev Inst Med Trop Sao Paulo. 1992;34(6):543-8. PubMed PMID: 1342123.

13. Coura-Filho P, Mendes NM, de Souza CP, Pereira JP. The prolonged use of niclosamide as a molluscicide for the control of *Schistosoma mansoni*. Rev Inst Med Trop Sao Paulo. 1992;34(5):427-31. PubMed PMID: 1342106.

14. Crossland NO. A mud-sampling technique for the study of the ecology of aquatic snails, and its use in the evaluation of the efficacy of molluscicides in field trials. Bull World Health Organ. 1962;27:125-33. PubMed PMID: 13882393; PubMed Central PMCID: PMC2555810.

15. Crossland NO. A large-scale experiment in the control of aquatic snails by the use of molluscicides on a sugar estate in the northern region of Tanganyika. Bull World Health Organ. 1963;29:515-24.

16. Crossland NO. Field trials to evaluate the effectiveness of the molluscicide N-tritylmorpholine in irrigation systems. Bull World Health Organ. 1967;37(1):23-42. PubMed PMID: 5300051; PubMed Central PMCID: PMC2554210.

17. de Souza CP, Mendes NM. [Repopulation of breeding habitats of *Biomphalaria glabrata* after treatment with niclosamide]. Rev Inst Med Trop Sao Paulo. 1991;33(4):297-302. PubMed PMID: 1844951.

18. Douglas PT. The control of *Schistosoma haematobium* in Kenya using molluscicide [MSc thesis]. London UK: The London School of Hygiene and Tropical Medicine; 2001.

19. Duke BO, Moore PJ. The use of a molluscicide in conjunction with chemotherapy to control *Schistosoma haematobium* at the Barombi Lake foci in Cameroon. II. Urinary examination methods, the use of niridazole to attack the parasite in man, and the effect on transmission from man to snail. Tropenmed Parasitol. 1976;27(4):489-504. PubMed PMID: 1006804.

20. Evans AC. Control of schistosomiasis in large irrigation schemes by use of niclosamide. A ten-year study in Zimbabwe. Am J Trop Med Hyg. 1983;32(5):1029-39. PubMed PMID: 6625058.

21. Farooq M, Hairston NG, Samaan SA. The effect of area-wide snail control on the endemicity of bilharziasis in Egypt. Bull World Health Organ. 1966;35(3):369-75. PubMed PMID: 5297632; PubMed Central PMCID: PMC2476090.

22. Fenwick A. The development of snail control methods on an irrigated sugar-cane estate in northern Tanzania. Bull World Health Organ. 1970;42(4):589-96. PubMed PMID: 5310954; PubMed Central PMCID: PMC2427453.

23. Fenwick A. Effect of a control programme on transmission of *Schistosoma mansoni* on an irrigated estate in Tanzania. Bull World Health Organ. 1972;47(3):325-30. PubMed PMID: 4539820; PubMed Central PMCID: PMC2480730.

24. Fenwick A, Jorgensen TA. The effect of a control programme against *Schistosoma mansoni* on the prevalence and intensity of infection on an irrigated sugar estate in northern Tanzania. Bull World Health Organ. 1972;47(5):579-86. PubMed PMID: 4540677; PubMed Central PMCID: PMC2480825.

25. Ferguson FF, Palmer JR, Jobin WR. Control of schistosomiasis on Vieques Island, Puerto Rico. Am J Trop Med Hyg. 1968;17(6):858-63. PubMed PMID: 5749745.

26. Foster R, Teesdale C, Poulton GF. Trials with a new molluscicide. Bull World Health Organ. 1960;22(5):543-8. PubMed PMID: 20604060; PubMed Central PMCID: PMC2555477.

27. Gilles HM, Abdel-Aziz Zaki A, Soussa MH, Samaan SA, Soliman Soliman S, Hassan A, et al. Results of a seven year snail control project on the endemicity of *Schistosoma haematobium* infection in Egypt. Ann Trop Med Parasitol. 1973;67(1):45-65. PubMed PMID: 4723214.

28. Goll PH, Lemma A, Duncan J, Mazengia B. Control of schistosomiasis in Adwa, Ethiopia, using the plant molluscicide endod (*Phytolacca dodecandra*). Tropenmed Parasitol. 1983;34(3):177-83. PubMed PMID: 6636299.

29. Goll PH, Wilkins HA, Marshall TF. Dynamics of *Schistosoma haematobium* infection in a Gambian community. II. The effect on transmission of the control of *Bulinus senegalensis* by the use of niclosamide. Trans R Soc Trop Med Hyg. 1984;78(2):222-6.

30. Gönnert R. Results of laboratory and field trials with the molluscicide Bayer 73. Bull World Health Organ. 1961;25:483-501. PubMed PMID: 13899472; PubMed Central PMCID: PMC2555706.

31. Greer GJ, Tchounwou PB, Takougang I, Monkiedje A. Field tests of a village-based mollusciciding programme for the control of snail hosts of human schistosomes in Cameroon. Trop Med Int Health. 1996;1(3):320-7. PubMed PMID: 8673834.

32. Gundersen SG, Birrie H, Torvik HP, Scherbaum H. Control of *Schistosoma mansoni* in the Blue Nile Valley of western Ethiopia by mass chemotherapy and focal snail control: a primary health care experience. Trans R Soc Trop Med Hyg. 1990;84(6):819-25. PubMed PMID: 2128985.

33. Jobin WR, Unrau GO. Chemical control of *Australorbis glabratus*. Public Health Rep. 1967;82(1):63-71. PubMed PMID: 4959383; PubMed Central PMCID: PMC1919867.

34. Jobin WR, Ferguson FF, Palmer JR. Control of schistosomiasis in Guayama and Arroyo, Puerto Rico. Bull World Health Organ. 1970;42(1):151-6. PubMed PMID: 5309511; PubMed Central PMCID: PMC2427502.

35. Jordan P, Barnish G, Bartholomew RK, Grist E, Christie JD. Evaluation of an experimental mollusciciding programme to control *Schistosoma mansoni* transmission in St Lucia. Bull World Health Organ. 1978;56(1):139-46. PubMed PMID: 307443; PubMed Central PMCID: PMC2395549.

36. Jordan P, Cook JA, Bartholomew RK, Grist E, Auguste E. *Schistosoma mansoni* control in Cul de Sac Valley, Saint Lucia. II. Chemotherapy as a supplement to a focal mollusciciding programme. Trans R Soc Trop Med Hyg. 1980;74(4):493-500. PubMed PMID: 7445046.

37. Kariuki HC, Madsen H, Ouma JH, Butterworth AE, Dunne DW, Booth M, et al. Long term study on the effect of mollusciciding with niclosamide in stream habitats on the transmission of schistosomiasis mansoni after community-based chemotherapy in Makueni District, Kenya. Parasit Vectors. 2013;6(1):107. doi: 10.1186/1756-3305-6-107. PubMed PMID: 23596985; PubMed Central PMCID: PMC3652733.

38. Lyons GR. Schistosomiasis in north-western Ghana. Bull World Health Organ. 1974;51(6):621-32. PubMed PMID: 4549612; PubMed Central PMCID: PMC2366268.

39. Macdonald F, Clarke Vde V, Gaddie P, Atkinson G. Report on a large-scale attempt at control of bilharziasis by combined mass treatment and intensive snail control. Cent Afr J Med. 1973;19:22-32. PubMed PMID: 4795849.

40. Madsen H, Rohde R, Maiga AS. Trials on focal molluscicide application in larger irrigation canals and lakes in Mali. Trop Med Parasitol. 1986;37(1):22-4. PubMed PMID: 3704470.

41. McCullough FS. The seasonal density of populations of *Bulinus (Physopsis) globosus* and *B. forskalii* in natural habitats in Ghana. Ann Trop Med Parasitol. 1957;51(3):235-48. PubMed PMID: 13470762.

42. Meyer-Lassen J, Daffalla AA, Madsen H. Evaluation of focal mollusciciding in the Rahad Irrigation Scheme, Sudan. Acta Trop. 1994;58(3-4):229-41. PubMed PMID: 7709862.

43. Paulini E, Chaia G, de FJ. Trials with the molluscicides Rhodiacid and Bayer 73. Bull World Health Organ. 1961;25:706-9. PubMed PMID: 14484505; PubMed Central PMCID: PMC2555721.

44. Pieri OS, Goncalves JF, Sarquis O. Repeated focal mollusciciding for snail control in a sugar-cane area of northeast Brazil. Mem Inst Oswaldo Cruz. 1995;90(4):535-6. PubMed PMID: 8551961.

45. Pitchford RJ. Findings in relation to schistosome transmission in the field following the introduction of various control measures. S Afr Med J. 1966;40 Suppl(36):3-16. PubMed PMID: 5920426.

46. Prentice MA, Barnish G. Granule formulations of molluscicides for use in developing countries. Ann Trop Med Parasitol. 1980;74(1):45-51. PubMed PMID: 7377873.

47. Prentice MA, Jordan P, Bartholomew RK, Grist E. Reduction in transmission of *Schistosoma mansoni* by a four-year focal mollusciciding programme against *Biomphalaria glabrata* in Saint Lucia. Trans R Soc Trop Med Hyg. 1981;75(6):789-98. PubMed PMID: 7330940.

48. Rosenfield PL, Smith RA, Wolman MG. Development and verification of a schistosomiasis transmission model. Am J Trop Med Hyg. 1977;26(3):505-16.

49. Saladin B, Saladin K, Holzer B, Dennis E, Hanson A, Degremont A. A pilot control trial of schistosomiasis in central Liberia by mass chemotherapy of target populations, combined with focal application of molluscicide. Acta Trop. 1983;40(3):271-95. PubMed PMID: 6138977.

50. Shiff CJ. Trials with a new molluscicide, Bayer 73, in Southern Rhodesia. Bull World Health Organ. 1961;25:533-42. PubMed PMID: 13911864; PubMed Central PMCID: PMC2555713.

51. Shiff CJ, Clarke Vde V, Evans AC, Barnish G. Molluscicide for the control of schistosomiasis in irrigation schemes: a study in Southern Rhodesia. Bull World Health Organ. 1973;48(3):299-307. PubMed PMID: 4542798; PubMed Central PMCID: PMC2481066.

52. Shiff CJ, Coutts WC, Yiannakis C, Holmes RW. Seasonal patterns in the transmission of *Schistosoma haematobium* in Rhodesia, and its control by winter application of molluscicide. Trans R Soc Trop Med Hyg. 1979;73(4):375-80. PubMed PMID: 555062.

53. Sturrock RF, Barnish G, Upatham ES. Snail findings from an experimental mollusciciding programme to control *Schistosoma mansoni* transmission in St. Lucia. International Journal of Parasitology. 1974;4:231-40.

54. Sturrock RF, Diaw OT, Talla I, Niang M, Piau JP, Capron A. Seasonality in the transmission of schistosomiasis and in populations of its snail intermediate hosts in and around a sugar irrigation scheme at Richard Toll, Senegal. Parasitology. 2001;123 Suppl:S77-89. PubMed PMID: 11769294.

55. Takougang I, Meli J, Angwafo F, 3rd. Field trials of low dose Bayluscide on snail hosts of schistosome and selected non-target organisms in sahelian Cameroon. Mem Inst Oswaldo Cruz. 2006;101(4):355-8. PubMed PMID: 16951803.

56. Tameim O, Zakaria ZB, Hussein H, el Gaddal AA, Jobin WR. Control of schistosomiasis in the new Rahad Irrigation Scheme of Central Sudan. J Trop Med Hyg. 1985;88(2):115-24. PubMed PMID: 4032520.

57. Webbe G. Laboratory and field trials of a new molluscicide, Bayer 73, in Tanganyika. Bull World Health Organ. 1961;25:525-31. PubMed PMID: 14005427; PubMed Central PMCID: PMC2555727.

58. Webbe G. The transmission of *Schistosoma haematobium* in an area of Lake Province, Tanganyika. Bull World Health Organ. 1962;27:59-85. PubMed PMID: 14005428; PubMed Central PMCID: PMC2555818.

59. Webbe G. Natural trends in snail populations in relation to control of bilharziasis in East Africa. East Afr Med J. 1965;42(11):605-13. PubMed PMID: 5864876.

60. Webbe G, el Hak S. Progress in the control of schistosomiasis in Egypt 1985-1988. Trans R Soc Trop Med Hyg. 1990;84(3):394-400. PubMed PMID: 2124392.

61. Werler C. Efficiency of focal molluscicide treatment against schistosomiasis reinfection in an irrigation scheme and in a small dams area in Mali. Preliminary communication. Tropenmed Parasitol. 1989;40(2):234-6. PubMed PMID: 2505385.

62. Wright WH, Dobrovolny CG, Berry EG. Field trials of various molluscicides (chiefly sodium pentachlorophenate) for the control of aquatic intermediate hosts of human bilharziasis. Bull World Health Organ. 1958;18(5-6):963-74. PubMed PMID: 13573120; PubMed Central PMCID: PMC2537948.

63. Zaki AA. The effect of systematic application of bayluscide on controlling bilharziasis. East Afr Med J. 1971;48(5):218-27. PubMed PMID: 5136916.
