# Supplementary material for: Systematic Review and Meta-analysis of the Impact of Chemical-Based Mollusciciding for Control of Schistosoma mansoni and S. haematobium Transmission
Source: PLoS Negl Trop Dis. 2015 Dec 28;9(12):e0004290. doi: 10.1371/journal.pntd.0004290 (PMC4692485; doi:10.1371/journal.pntd.0004290)
Supplement: S4 File — Papers reviewed but not included in the qualitative or quantitative analyses. (DOCX) [file pntd.0004290.s012.docx]

Papers reviewed but not included in the qualitative of quantitative analysis.

1. al-Madani AA. Problems in the control of schistosomiasis in Asir Province, Saudi Arabia. Journal of community health. 1991;16(3):143-9. PubMed PMID: 1860966.

2. Amazigo UO, Anago-Amanze CI, Okeibunor JC. Urinary schistosomiasis among school children in Nigeria: consequences of indigenous beliefs and water contact activities. J Biosoc Sci. 1997;29(1):9-18. PubMed PMID: 9881116.

3. Amin AH, Fenwick A. The control of snails on a small scale. Tropical Doctor. 1978;8(1):8-12. PubMed PMID: 628947.

4. Andrews P, Thyssen J, Lorke D. The biology and toxicology of molluscicides, Bayluscide. Pharmacology & Therapeutics. 1983;19(2):245-95. PubMed PMID: 6763710.

5. Arfaa F, Bijan H, Farahmandian I. Present status of urinary bilharziasis in Iran. Trans R Soc Trop Med Hyg. 1967;61:358-67.

6. Arfaa F, Farahmandian I, Soleimani M. Evaluation of the effect of mass chemotherapy with niridazole as a method of bilharziasis control in Iran. Trans R Soc Trop Med Hyg. 1970;64(1):130-3. PubMed PMID: 5442031.

7. Arijo AG, Qureshi TA, Pathan ZA. A studies on chemical control of *S. mansoni* intermediate host. Pakistan Journal of Biological Sciences: PJBS. 2007;10(15):2606-8. PubMed PMID: 19070141.

8. Bagalwa M, Baluku B. [Monthly variations in the level of infestation and the potential for transmission of *Biomphalaria pfeifferi* in 2 aquatic systems in Lwiro, Democratic Republic of Congo]. Medecine Tropicale 1998;58(4):372-4. PubMed PMID: 10399696.

9. Barakat RMR. Epidemiology of schistosomiasis in Egypt: Travel through time: Review. Journal of Advanced Research. 2013;4:425-32. Epub 5 September 2012. doi: <http://dx.doi.org/10.1016/j.jare.2012.07.003>.

10. Barbosa FS, Pinto R, Souza OA. Control of schistosomiasis mansoni in a small north east Brazilian community. Trans R Soc Trop Med Hyg. 1971;65(2):206-13. PubMed PMID: 5104969.

11. Barnish G. The control of bilharziasis by the use of molluscicides. The Central African Journal of Medicine. 1970:Suppl:22-7. PubMed PMID: 5471343.

12. Barnish G. Evaluation of chemotherapy in the control of *Schistosoma mansoni* in Marquis Valley, Saint Lucia. II. Biological results. The American Journal of Tropical Medicine and Hygiene. 1982;31(1):111-5. PubMed PMID: 7058972.

13. Barnish G, Christie JD, Prentice MA. *Schistosoma mansoni* control in Cul de Sac Valley, Saint Lucia. I. A two-year focal surveillance-mollusciciding programme for the control of *Biomphalaria glabrata*. Trans R Soc Trop Med Hyg. 1980;74(4):488-92. PubMed PMID: 7445045.

14. Barnish G, Prentice MA. Lack of resistance of the snail *Biomphalaria glabrata* after nine years of exposure to Bayluscide. Trans R Soc Trop Med Hyg. 1981;75(1):106-7. PubMed PMID: 7268836.

15. Barnish G, Prentice MA. Predation of the snail *Biomphalaria glabrata* by freshwater shrimps in St. Lucia, West Indies. Annals of Tropical Medicine and Parasitology. 1982;76(1):117-20. PubMed PMID: 7082076.

16. Barnish G, Sturrock RF. Letter: Aerial application of a molluscicide to a marsh. Trans R Soc Trop Med Hyg. 1973;67(4):610-1. PubMed PMID: 4785468.

17. Bolton P. Schistosomiasis control in irrigation schemes in Zimbabwe. The Journal of Tropical Medicine and Hygiene. 1988;91(3):107-14. PubMed PMID: 3392763.

18. Boyce CB, Tieze-Dagevos JW, Larman VN. The susceptibility of *Biomphalaria glabrata* throughout its life-history to N-tritylmorpholine. Bulletin of the World Health Organization. 1967;37(1):13-21. PubMed PMID: 5300047; PubMed Central PMCID: PMC2554225.

19. Brinkmann UK, Werler C, Traore M, Korte R. The costs of schistosomiasis control in a Sahelian country. Tropenmed Parasitol. 1988;39(2):175-81. PubMed PMID: 3140360.

20. Chandiwana SK. Community water-contact patterns and the transmission of *Schistosoma haematobium* in the highveld region of Zimbabwe. Social Science & Medicine. 1987;25(5):495-505. PubMed PMID: 3118479.

21. Chandiwana SK, Taylor P, Chimbari M, Ndhlovu P, Makura O, Bradley M, et al. Control of schistosomiasis transmission in newly established smallholder irrigation schemes. Trans R Soc Trop Med Hyg. 1988;82(6):874-80. PubMed PMID: 3151528.

22. Chernin E, Michelson EH, Augustine DL. Studies on the biological control of schistosome-bearing snails. I. The control of *Australorbis glabratus* populations by the snail, *Marisa cornuarietis*, under laboratory conditions. The American Journal of Tropical Medicine and Hygiene. 1956;5(2):297-307. PubMed PMID: 13302628.

23. Christie JD, Prentice MA, Barnish G. Control of schistosomiasis by mollusciciding. The American Journal of Tropical Medicine and Hygiene. 1980;29(2):323-4. PubMed PMID: 7369453.

24. Chu KY, Vanderburg JA, Klumpp RK. Transmission dynamics of miracidia of *Schistosoma haematobium* in the Volta Lake. Bulletin of the World Health Organization. 1981;59(4):555-60. PubMed PMID: 6976225; PubMed Central PMCID: PMC2396088.

25. Clarke VD, Shiff CJ, Blair DM. The control of snail hosts of bilharziasis and fascioliasis in Southern Rhodesia. Bulletin of the World Health Organization. 1961;25:549-58. PubMed PMID: 13879773; PubMed Central PMCID: PMC2555739.

26. Crossland NO. The pest status and control of the tadpole shrimp, *Triops granarius,* and of the snail, *Lanistes ovum,* in Swaziland rice fields. Journal of Applied Ecology. 1964;2:115-20.

27. Dawood IK, Dazo BC, Farooq M. Large-scale application of Bayluscide and sodium pentachlorophenate in the Egypt-49 project area. Evaluation of relative efficacy and comparative costs. Bulletin of the World Health Organization. 1966;35(3):357-67. PubMed PMID: 5297631; PubMed Central PMCID: PMC2476088.

28. Dawson VK. Environmental fate and effects of the lampricide Bayluscide: a review. J Great Lakes Res. 2003;29(Supplement 1):475-92.

29. de Lima e Costa MF, Rocha RS, Coura Filho P, Katz N. A 13-year follow-up of treatment and snail control in an area endemic for *Schistosoma mansoni* in Brazil: incidence of infection and reinfection. Bulletin of the World Health Organization. 1993;71(2):197-205. PubMed PMID: 8490983; PubMed Central PMCID: PMC2393443.

30. Duke BO, Moore PJ. The control of *Schistosoma haematobium* in West Cameroon. Trans R Soc Trop Med Hyg. 1971;65(6):841-3. PubMed PMID: 5157446.

31. Duke BO, Moore PJ. The use of a molluscicide in conjunction with chemotherapy to control *Schistosoma haematobium* at the Barombi Lake foci in Cameroon. III. Conclusions and costs. Tropenmed Parasitol. 1976;27(4):505-8. PubMed PMID: 1006805.

32. Duke BO, Moore PJ. The use of a molluscicide, in conjunction with chemotherapy, to control *Schistosoma haematobium* at the Barombi Lake foci in Cameroon. I. The attack on the snail hosts, using N-tritylmorpholine, and the effect on transmission from snail to man. Tropenmed Parasitol. 1976;27(3):297-313. PubMed PMID: 982548.

33. el Gaddal AA. The Blue Nile Health Project: a comprehensive approach to the prevention and control of water-associated diseases in irrigated schemes of the Sudan. The Journal of Tropical Medicine and Hygiene. 1985;88(2):47-56. PubMed PMID: 4032529.

34. Engels D, Ndoricimpa J, Gryseels B. Schistosomiasis mansoni in Burundi: progress in its control since 1985. Bulletin of the World Health Organization. 1993;71(2):207-14. PubMed PMID: 8490984; PubMed Central PMCID: PMC2393458

35. Farooq M. Pre-control investigations in bilharziasis. The Journal of Tropical Medicine and Hygiene. 1969;72(1):14-8. PubMed PMID: 5813099.

36. Farringer JE. The determination of the acute toxicity of Rotenone and Bayer 73 to selected aquatic organisms. [Thesis] La Crosse, Wisconsin: University of Wisconsin; 1972.

37. Fenwick A, Cheesmond AK, Amin MA. The role of field irrigation canals in the transmission of *Schistosoma mansoni* in the Gezira Scheme, Sudan. Bulletin of the World Health Organization. 1981;59(5):777-86. PubMed PMID: 6976239; PubMed Central PMCID: PMC2396103.

38. Fenwick A, Lidgate HJ. Attempts to eradicate snails from impounded water by the use of N-tritylmorpholine. Bulletin of the World Health Organization. 1970;42(4):581-8. PubMed PMID: 5310953; PubMed Central PMCID: PMC2427465.

39. Foster R. Schistosomiasis on an irrigated estate in East Africa. 2. Epidemiology. The Journal of Tropical Medicine and Hygiene. 1967;70(7):159-68. PubMed PMID: 6067571.

40. Gamet A, Brottes H, Mvogo L. [Preliminary experiments in the control of bilharziasis vectors in the ponds of pisciculture station in the Cameroons]. Bulletin de la Societe de Pathologie Exotique et de ses Filiales. 1964;57:118-24. PubMed PMID: 14185697.

41. Gillet J, Bruaux P. [Laboratory trials of the new molluscicides Bayer 73 and ICI 24223]. Bulletin of the World Health Organization. 1961;25:509-17. PubMed PMID: 13898641; PubMed Central PMCID: PMC2555724.

42. Gillet J, Bruaux P. Laboratory and field testing of Bayluscide (Bayer 73). Pflanzen-schutz-Nachrichten Bayer. 1962;15:70-4.

43. Giovanelli A, Silva CL, Medeiros L, Vasconcellos MC. The molluscicidal activity of niclosamide (Bayluscide WP70(R)) on *Melanoides tuberculata* (Thiaridae), a snail associated with habitats of *Biomphalaria glabrata* (Planorbidae). Memorias do Instituto Oswaldo Cruz. 2002;97(5):743-5. PubMed PMID: 12219145.

44. Gönnert R. Experiences in the control of bilharziasis with molluscicides. Annales des Societes Belges de Medecine Tropicale, de Parasitologie, et de Mycologie. 1967;47(2):195-203. PubMed PMID: 5596722.

45. Greer GJ, Mimpfoundi R, Malek EA, Joky A, Ngonseu E, Ratard RC. Human schistosomiasis in Cameroon. II. Distribution of the snail hosts. The American Journal of Tropical Medicine and Hygiene. 1990;42(6):573-80. PubMed PMID: 2372088.

46. Hairston NG. An analysis of age-prevalence data by catalytic models. A contribution to the study of bilharziasis. Bulletin of the World Health Organization. 1965;33(2):163-75.

47. Harrison AD. The effects of Bayluscid on gastropod snails and other aquatic fauna in Rhodesia. Hydrobiologia. 1966;28:371-84.

48. Harrison AD, Mason MH. The effects on the fauna of natural waters of surveillance treatment with Bayluscide in Rhodesia. Hydrobiologia. 1967;29:149-55.

49. Highton RB, Choudhry AW. The cost evaluation of mollusciciding operations on five irrigation schemes in Kenya. East African Medical Journal. 1974;51(2):180-93. PubMed PMID: 4837254.

50. Hira PR. Aspects of the spread and control of schistosomiasis in Ibadan, Nigeria. The West African Medical Journal and Nigerian Practitioner. 1970;19(6):180-3. PubMed PMID: 5533674.

51. Hofkin BV, Stryker GA, Koech DK, Loker ES. Consumption of *Biomphalaria glabrata* egg masses and juveniles by the ampullariid snails *Pila ovata*, *Lanistes carinatus* and *Marisa cornuarietis*. Acta Tropica. 1991;49(1):37-44. PubMed PMID: 1678574.

52. Jobin WR. Rationale for selecting molluscicides for bilharzia control programs. Public Health Reports. 1968;83(7):594-6. PubMed PMID: 4969690; PubMed Central PMCID: PMC1891876.

53. Jobin WR. Economics of the application of molluscicides to flowing water. Bulletin of the World Health Organization. 1968;38(2):322-3. PubMed PMID: 5302307; PubMed Central PMCID: PMC2554324.

54. Jobin WR. Population dynamics of aquatic snails in three farm ponds of Puerto Rico. The American Journal of Tropical Medicine and Hygiene. 1970;19(6):1038-48. PubMed PMID: 5493050.

55. Jobin WR. Control of *Biomphalaria glabrata* in a small reservoir by fluctuation of the water level. The American Journal of Tropical Medicine and Hygiene. 1970;19(6):1049-54. PubMed PMID: 5493051.

56. Jobin WR. Cost of snail control. The American Journal of Tropical Medicine and Hygiene. 1979;28(1):142-54. PubMed PMID: 107817.

57. Jordan P, Christie JD, Unrau GO. Schistosomiasis transmission with particular reference to possible ecological and biological methods of control. A review. Acta Tropica. 1980;37(2):95-135. PubMed PMID: 6106355.

58. Jurberg P. Why it is difficult to control *Biomphalaria glabrata,* the vector snail of schistosomiasis. Memorias do Instituto Oswaldo Cruz. 1987;82 Suppl 4:203-7. PubMed PMID: 3151094.

59. Khallaayoune K, Madsen H, Laamrani H. Evaluation of three methods to control *Bulinus truncatus*, the intermediate host of *Schistosoma haematobium* in an irrigation scheme, Tessaout-Amont, Morocco. Acta Tropica. 1998;69(1):51-63. PubMed PMID: 9588241.

60. Klumpp RK, Chu KY. Ecological studies of *Bulinus rohlfsi*, the intermediate host of *Schistosoma haematobium* in the Volta Lake. Bulletin of the World Health Organization. 1977;55(6):715-30. PubMed PMID: 304396; PubMed Central PMCID: PMC2366721.

61. Korte R, Schmidt-Ehry B, Kielmann AA, Brinkmann UK. Cost and effectiveness of different approaches to schistosomiasis control in Africa. Tropenmed Parasitol. 1986;37(2):149-52. PubMed PMID: 3092332.

62. Laamrani H, Khallaayoune K, Boelee E, Laghroubi MM, Madsen H, Gryseels B. Evaluation of environmental methods to control snails in an irrigation system in Central Morocco. Tropical Medicine & International Health : TM & IH. 2000;5(8):545-52.

63. Levine ND. Integrated control of snails. American Zoologist. 1970;10(4):579-82. PubMed PMID: 5530745.

64. Lima e Costa MF, Guerra HL, Pimenta FG, Jr., Firmo JO, Uchoa E. [Evaluation of a program for the control of schistosomiasis in municipalities located on the basin of the Sao Francisco River, Minas Gerais, Brazil]. Revista da Sociedade Brasileira de Medicina Tropical. 1996;29(2):117-26. PubMed PMID: 8713603.

65. Machado PA. The Brazilian program for schistosomiasis control, 1975-1979. The American Journal of Tropical Medicine and Hygiene. 1982;31(1):76-86. PubMed PMID: 7199262.

66. Mandahl-Barth G. Key to the identification of east and central African freshwater snails of medical and veterinary importance. Bulletin of the World Health Organization. 1962;27:135-50. PubMed PMID: 14469160; PubMed Central PMCID: PMC2555811.

67. Marking LL, Hogan JW. Toxicity of Bayer 73 to fish. In: Fish and Wildlife Service, editor. Washington: US Department of the Interior; 1967.

68. Massoud J, Chu KY, Arfaa F. Field trials of Bayluscide, sodium pentachlorophenate and copper sulphate in standing waters in Iran. Annals of Tropical Medicine and Parasitology. 1969;63(2):189-94. PubMed PMID: 5372136.

69. McCullough F. Snail control in relation to a strategy for reduction of morbidity due to schistosomiasis. Tropenmed Parasitol. 1986;37(2):181-4. PubMed PMID: 3092336.

70. McCullough FS. The distribution of human schistosomiasis and the potential snail hosts in Ghana. The West African Medical Journal. 1957;6(3):87-97. PubMed PMID: 13468370.

71. McCullough FS. A preliminary note on the degree of compatibility between *Schistosoma haematobium* and its bulinid vectors. The West African Medical Journal. 1957;6(3):98-100. PubMed PMID: 13468371.

72. McCullough FS. The susceptibility and resistance of *Bulinus (Physopsis) globosus* and *Bulinus (Bulinus) truncatus* *rohlfsi* to two strains of *Schistosoma haematobium* in Ghana. Bulletin of the World Health Organization. 1959;20(1):75-85. PubMed PMID: 13638791; PubMed Central PMCID: PMC2537803.

73. McCullough FS. Biological control of the snail intermediate hosts of human *Schistosoma* spp.: a review of its present status and future prospects. Acta Tropica. 1981;38(1):5-13. PubMed PMID: 6111917.

74. McCullough FS, Gayral P, Duncan J, Christie JD. Molluscicides in schistosomiasis control. Bulletin of the World Health Organization. 1980;58(5):681-9. PubMed PMID: 6975179.

75. McMullen DB, Buzo ZJ, Rainey MB, Francotte J. Bilharziasis control in relation to water resources development in Africa and the Middle East. Bulletin of the World Health Organization. 1962;27(1):25-40. PubMed PMID: 20604119; PubMed Central PMCID: PMC2555824.

76. Meyling AH, Schutte CH, Pitchford RJ. Some laboratory investigations on Bayer 73 and ICI 24223 as molluscicides. Bulletin of the World Health Organization. 1962;27:95-8. PubMed PMID: 14473215; PubMed Central PMCID: PMC2555812.

77. Morley NJ, Irwin SW, Lewis JW. Pollution toxicity to the transmission of larval digeneans through their molluscan hosts. Parasitology. 2003;126 Suppl:S5-26. PubMed PMID: 14667169.

78. Mozley A. Molluscicides. London: H.K. Lewis & Co.; 1952.

79. Mozley A. Sites of Infection: Unstable Areas as Sources of Parasitic Diseases: Schistosomiasis and Fascioliasis. London: H.K. Lewis & Co.; 1955. 86 p.

80. Palmer JR, Colon AZ, Ferguson FF, Jobin WR. The control of schistosomiasis in Patillas, Puerto Rico. Public health reports. 1969;84(11):1003-7. PubMed PMID: 4982247; PubMed Central PMCID: PMC2031691.

81. Pesigan TP, Hairston NG. The effect of snail control on the prevalence of *Schistosoma japonicum* infection in the Philippines. Bulletin of the World Health Organization. 1961;25:479-82. PubMed PMID: 14485669; PubMed Central PMCID: PMC2555707.

82. Pieri OS, Barbosa CS, Moza PG. Schistosomiasis control based on repeated chemotherapy in a rural village of the sugar-cane zone in northeast Brazil. Memorias do Instituto Oswaldo Cruz. 1998;93 Suppl 1:259-64. PubMed PMID: 9921364.

83. Pointier JP, Giboda M. The case for biological control of snail intermediate hosts of *Schistosoma mansoni*. Parasitology Today. 1999;15(10):395-7. PubMed PMID: 10481149.

84. Polderman AM. Cost-effectiveness of different ways of controlling intestinal schistosomiasis: a case study. Social Science & Medicine. 1984;19(10):1073-80. PubMed PMID: 6441261.

85. Ritchie LS, Berrios-Duran LA, Frick LP, Fox I. Molluscicidal qualities of Bayluscide (Bayer 73) revealed by 6-hour and 24-hour exposures against representative stages and sizes of *Australorbis glabratus*. Bulletin of the World Health Organization. 1963;29:281-6. PubMed PMID: 14056285; PubMed Central PMCID: PMC2554842.

86. Sarquis O, Pieri OS, dos Santos JA. Effects of Bayluscide WP 70 on the survival and water-leaving behaviour of Biomphalaria straminea, snail host of schistosomiasis in northeast Brazil. Memorias do Instituto Oswaldo Cruz. 1997;92(5):619-23. PubMed PMID: 9566228.

87. Scholefield RJ, Bergstedt RA, Bills TD. Relation of concentration and exposure time to the efficay of niclosamide against larval sea lampreys (*Petromyzon marinus*). J Great Lakes Res. 2003;29(Supplement 1):493-9.

88. Shiff CJ. The role of molluscicides in bilharzia control. South African Medical Journal. 1970;44(7):167-8. PubMed PMID: 5461625.

89. Shiff CJ. The value of incidence for the assessment of schistosomiasis control; a study in Southern Rhodesia. Bulletin of the World Health Organization. 1973;48(4):409-14. PubMed PMID: 4543546; PubMed Central PMCID: PMC2481102.

90. Shiff CJ, Garnett B. The short-term effects of three molluscicides on the microflora and microfauna of small, biologically stable ponds in Southern Rhodesia. Bulletin of the World Health Organization. 1961;25:543-7. PubMed PMID: 13911863; PubMed Central PMCID: PMC2555718.

91. Sleigh AC, Mott KE, Hoff R, Barreto ML, Mota EA, Maguire JH, et al. Three-year prospective study of the evolution of Manson's schistosomiasis in north-east Brazil. Lancet. 1985;2(8446):63-6. PubMed PMID: 2861524.

92. Souza CP. Molluscicide control of snail vectors of schistosomiasis. Memorias do Instituto Oswaldo Cruz. 1995;90(2):165-8. PubMed PMID: 8531652.

93. Sturrock RF. Field studies on the transmission of *Schistosoma mansoni* and on the bionomics of its intermediate host, *Biomphalaria glabrata*, on St. Lucia, West Indies. International Journal for Parasitology. 1973;3(2):175-94. PubMed PMID: 4706570.

94. Sturrock RF. Control of *Schistosoma mansoni* transmission: strategy for using molluscicides on St. Lucia. International Journal for Parasitology. 1973;3(6):795-801. PubMed PMID: 4762126.

95. Sturrock RF, Barnish G. The aerial application of molluscicides with special reference to schistosomiasis control. Bulletin of the World Health Organization. 1973;49(3):283-5. PubMed PMID: 4546522; PubMed Central PMCID: PMC2481155.

96. Takougang I, Meli J, Wabo Pone J, Angwafo F, 3rd. Community acceptability of the use of low-dose niclosamide (Bayluscide), as a molluscicide in the control of human schistosomiasis in Sahelian Cameroon. Annals of Tropical Medicine and Parasitology. 2007;101(6):479-86. doi: 10.1179/136485907X193833. PubMed PMID: 17716430.

97. Tameim O, Abdu KM, el Gaddal AA, Jobin WR. Protection of Sudanese irrigation workers from schistosome infections by a shift to earlier working hours. The Journal of Tropical Medicine and Hygiene. 1985;88(2):125-30. PubMed PMID: 4032521.

98. Tanaka H, Yasuraoka K, McCullough FS. Parameters used for assessment of molluscicides. The Japanese Journal of Experimental Medicine. 1986;56(4):189-94. PubMed PMID: 3820737.

99. Thomas JD. Schistosomiasis and control of molluscan hosts of human schistosomes with particular reference to possible self-regulatory mechanisms. Adv Parasitol. 1973;11:307-94.

100. Thomas JD, Tait AI. Control of the snail hosts of schistosomiasis by environmental manipulation: a field and laboratory appraisal in the Ibadan area, Nigeria. Philosophical transactions of the Royal Society of London Series B, Biological Sciences. 1984;305(1123):201-53. PubMed PMID: 6144118.

101. Webbe G. Control of transmission of *Schistosoma mansoni* (Sambon) in the Mirongo River. East African Medical Journal. 1964;41:508-20. PubMed PMID: 14249960.

102. Webbe G. Quantitative studies of intermediate host populations in the transmission of schistosomes. Proceedings of the Royal Society of Medicine. 1968;61(5):455. PubMed PMID: 5690275; PubMed Central PMCID: PMC1902505.

103. Webbe G, Msangi AS. Observations on three species of *Bulinus* on the east coast of Africa. Annals of Tropical Medicine and Parasitology. 1958;52(3):302-14. PubMed PMID: 13595557.

104. Webbe G, Sturrock RF. Laboratory tests of some new molluscicides in Tanganyika. Annals of Tropical Medicine and Parasitology. 1964;58:234-9. PubMed PMID: 14183113.

105. WHO. Molluscicide screening and evaluation. Bulletin of the World Health Organization. 1965;33(4):567-81. PubMed PMID: 5295001; PubMed Central PMCID: PMC2475882.

106. Woolhouse ME, Chandiwana SK. Spatial and temporal heterogeneity in the population dynamics of *Bulinus globosus* and *Biomphalaria pfeifferi* and in the epidemiology of their infection with schistosomes. Parasitology. 1989;98(Pt 1):21-34.

107. Woolhouse ME, Chandiwana SK. The epidemiology of schistosome infections of snails: taking the theory into the field. Parasitology Today. 1990;6(3):65-70. PubMed PMID: 15463299.

108. Woolhouse ME, Chandiwana SK. Population dynamics model for *Bulinus globosus*, intermediate host for *Schistosoma haematobium*, in river habitats. Acta Tropica. 1990;47(3):151-60.

109. Yang GJ, Li W, Sun LP, Wu F, Yang K, Huang YX, et al. Molluscicidal efficacies of different formulations of niclosamide: result of meta-analysis of Chinese literature. Parasites & Vectors. 2010;3:84. doi: 10.1186/1756-3305-3-84. PubMed PMID: 20819229; PubMed Central PMCID: PMC2944309.

110. Yang GJ, Sun LP, Hong QB, Zhu HR, Yang K, Gao Q, et al. Optimizing molluscicide treatment strategies in different control stages of schistosomiasis in the People's Republic of China. Parasites & Vectors. 2012;5:260. doi: 10.1186/1756-3305-5-260. PubMed PMID: 23151396; PubMed Central PMCID: PMC3533975.
