# Supplementary material for: Systematic Review and Meta-analysis of the Impact of Chemical-Based Mollusciciding for Control of Schistosoma mansoni and S. haematobium Transmission
Source: PLoS Negl Trop Dis. 2015 Dec 28;9(12):e0004290. doi: 10.1371/journal.pntd.0004290 (PMC4692485; doi:10.1371/journal.pntd.0004290)

I. Funnel plot and regression analysis of included studies reporting on impact of mollusciciding on human **prevalence** of *Schistosoma mansoni* or *S. haematobium* infection, N = 30

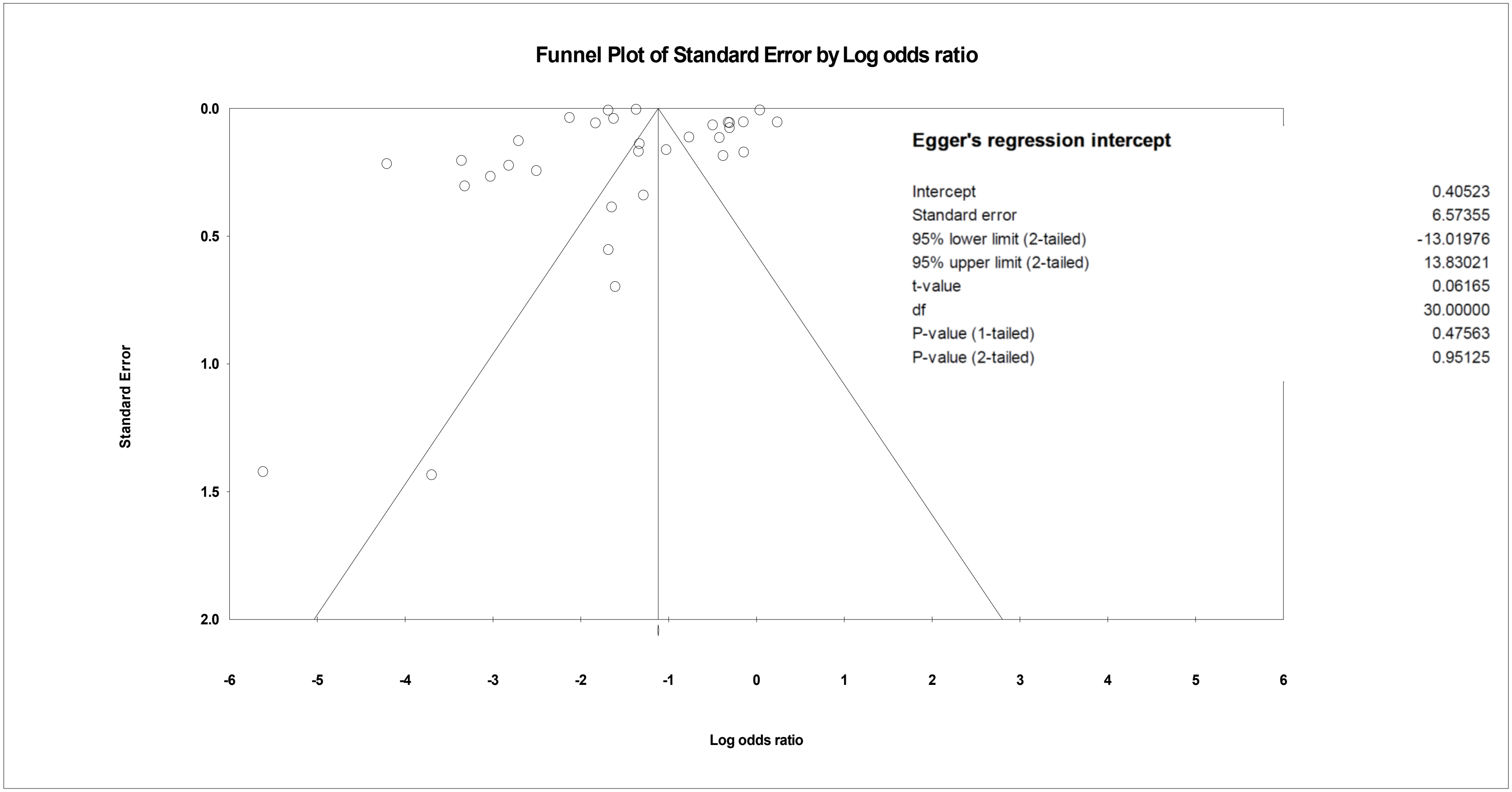

II. Funnel plot and regression analysis of included studies reporting on impact of mollusciciding on human **incidence** of *Schistosoma mansoni* or *S. haematobium* infection, N = 16

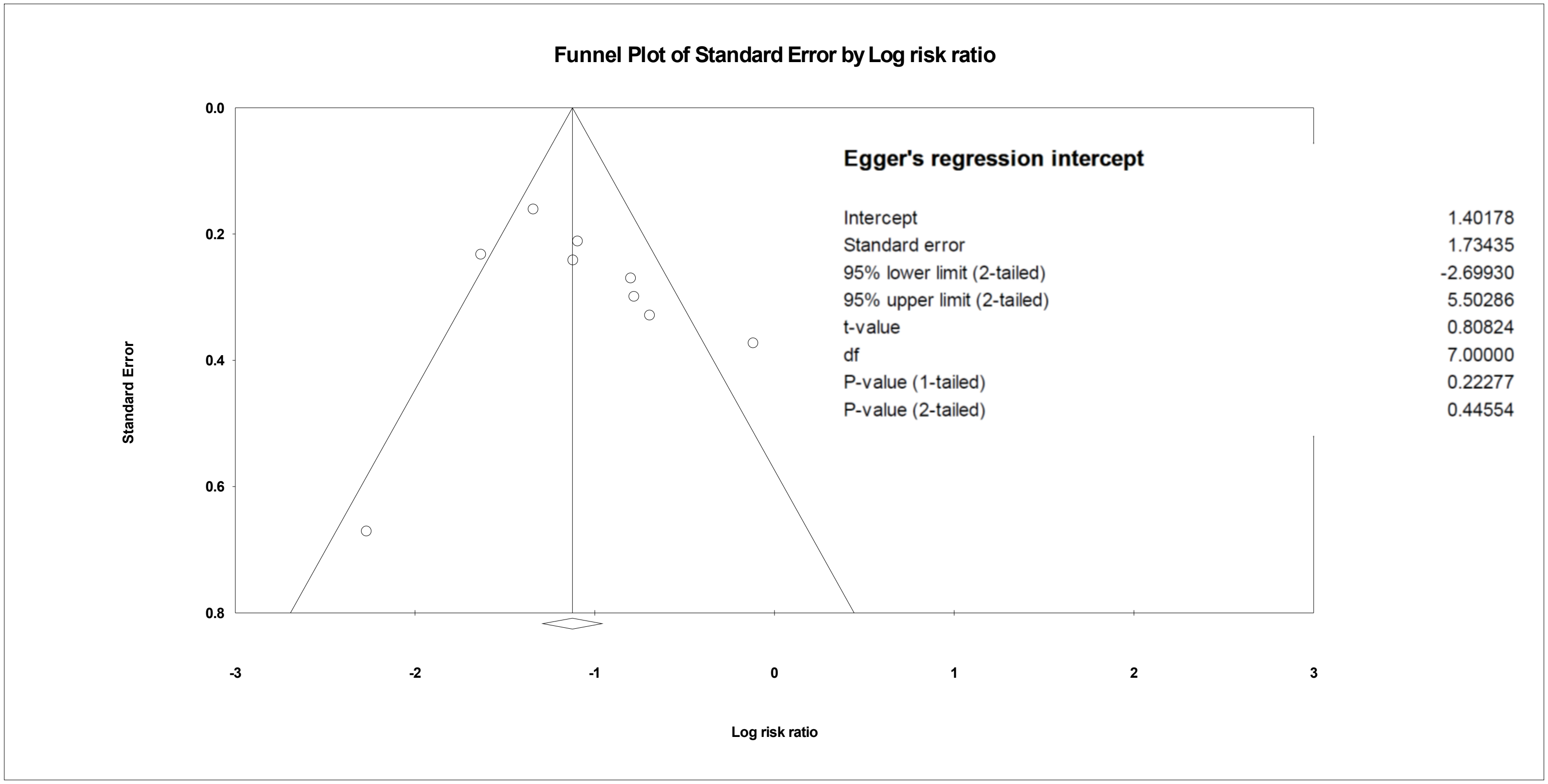

Supplement: S5 File — Graphical and regression analysis for potential publication bias of studies included in the meta-analysis, measuring, respectively, the impact of mollusciciding on local human prevalence (page 1) and incidence (page 2) of Schistosoma infection. (PDF) [file pntd.0004290.s013.pdf]
